# Molecular Characterization of a Novel Avian Influenza A (H2N9) Strain Isolated from Wild Duck in Korea in 2018

**DOI:** 10.3390/v11111046

**Published:** 2019-11-10

**Authors:** Seon-Ju Yeo, Duc-Duong Than, Hong-Seog Park, Haan Woo Sung, Hyun Park

**Affiliations:** 1 Zoonosis Research Center, Department of Infection Biology, School of Medicine, Wonkwang University, Iksan 54538, Korea; yeosj@wku.ac.kr (S.-J.Y.); ducduong27189@gmail.com (D.-D.T.); 2 GnCBio Inc, 4F, Yegan Plaza, 36, Banseok-ro, Yuseong-gu, Daejeon 34069, Korea; 5022daniel@naver.com; 3 College of Veterinary Medicine, Kangwon National University, Chuncheon 24341, Korea

**Keywords:** novel avian influenza virus isolate, H2N9, Korea, wild duck

## Abstract

A novel avian influenza virus (A/wild duck/Korea/K102/2018) (H2N9) was isolated from wild birds in South Korea in 2018, and phylogenetic and molecular analyses were conducted on complete gene sequences obtained by next-generation sequencing. Phylogenetic analysis indicated that the hemagglutinin (HA) and neuraminidase (NA) genes of the A/wild duck/Korea/K102/2018 (H2N9) virus belonged to the Eurasian countries, whereas other internal genes (polymerase basic protein 1 (PB1), PB2, nucleoprotein (NP), polymerase acidic protein (PA), matrix protein (M), and non-structural protein (NS)) belonged to the East Asian countries. A monobasic amino acid (PQIEPR/GLF) at the HA cleavage site, E627 in the PB2 gene, and no deletion of the stalk region in the NA gene indicated that the A/wild duck/Korea/K102/2018 (H2N9) isolate was a typical low pathogenicity avian influenza (LPAI). Nucleotide sequence similarity analysis of HA revealed that the highest homology (98.34%) is to that of A/duck/Mongolia/482/2015 (H2N3), and amino acid sequence of NA was closely related to that of A/duck/Bangladesh/8987/2010 (H10N9) (96.45%). In contrast, internal genes showed homology higher than 98% compared to those of other isolates derived from duck and wild birds of China or Japan in 2016–2018. The newly isolated A/wild duck/Korea/K102/2018 (H2N9) strain is the first reported avian influenza virus in Korea, and may have evolved from multiple genotypes in wild birds and ducks in Mongolia, China, and Japan.

## 1. Introduction

Influenza viruses are classified based on 18 hemagglutinin (HA) and 11 neuraminidase (NA) surface proteins into a large variety of subtypes, some of which are a public health threat [1]. Among these various subtypes, 16 subtypes of HA (H1–H16) and 9 subtypes of NA (N1–N9) belong to avian influenza viruses (AIV), while H17N10 and H18N11 subtypes are only detected in bats [2]. The wide circulation of AIVs in various hosts may lead to exchange of gene segments, which in turn leads to antigenic variation and formation of new AIVs. This can cause severe outbreaks and epidemics, resulting in huge economic losses to the poultry industry, and poses a serious threat to human health [3]. HA plays a central role in the life cycle of influenza A viruses through its involvement in receptor recognition, virus attachment, membrane fusion, and entry [4]. HA is a single-pass type I transmembrane glycoprotein present as a homotrimer on the viral surface that extends ~130 Å from the membrane. Each HA monomer contains two subunits, HA1 and HA2, produced by protease cleavage of the inactive precursor, HA0. The globular head of HA1 carries a shallow grove, a receptor-binding site (RBS), which is responsible for receptor recognition and host tropism [5]. 

The prevalence of H2 subtype AIV is much lower than that of other subtypes such as H5, H7, and H9, [6] but H2 influenza viruses are found in wild birds [7], swine [8], and humans. The Asian pandemic of H2N2 influenza A virus resulted from reassortment of previously circulating human H1N1 and avian H2N2 viruses from 1957 to 1968 [8]. In 2006, an H2N3 virus isolated from swine in the United States of America, belonging to the American AIV lineage, shared 84–85% similarity with the H2N2 viruses of the 1957 influenza pandemic [9,10]. In wild waterfowl birds, the H2 subtype was mainly identified in mallard ducks in comparison to other subtypes [7,11,12]. 

Based on three patients infected by human H7N9, the constructed phylogenetic trees of NA genes showed H7N9, H2N9, or H11N9 as its origin. However, the Korean H7 subtype was suggested as the origin of highly pathogenic avian influenza (HPAI) H7N9 2013 [13]. H7N9 AIV has all three of its genes reasserted: hemagglutinin (HA) gene derived from H7N3, NA gene potentially from H7N9, H2N9, or H11N9 and six internal genes derived from H9N2 viruses [13]. Furthermore, the pandemic human H2N2 subtype (1957) originated upon reassortment between a previously circulating seasonal human A/H1N1 virus and an avian A/H2N2 virus [14]. 

Therefore, continuous surveillance of H2 subtype AIV in Korea is imperative.

To date, H2N9 has been absent in Korea but avian LPAI H7N9 has been recently detected in migratory birds [15,16]. Furthermore, the Food and Agriculture Organization of the United Nations (FAO) assessed that the likelihood of H7N9 transmission is low with high uncertainty for the Russian Federation, Mongolia, Japan, and the Republic of Korea through movements of migratory or nomadic wild birds [17].

Therefore, continuous surveillance of H2N9 and LPAI H7N9 is highly recommended to predict the potential spread of HPAI H7N9 in Korea. Other subtypes (H7N3, H9N2, and H11N9), have been co-circulating in Korea [18,19,20]. The NA gene of the H7N9 influenza virus is more closely related to that of H2N9 viruses found in migratory wild birds in Hong Kong in 2010–2011 during the evolution of HPAI H7N9 [14]. 

For AIV surveillance in wild birds, AIV genomic analysis by next-generation sequencing (NGS) is ideal, as the mutation rate of influenza A viruses has been traditionally determined by sequencing different cDNA clones obtained from multiple plaques descending from a plaque-purified influenza A virus [21]. In addition, NGS has the advantage of allowing the sequencing of multiple gigabases of DNA in a single run, whereas RT-PCR amplification of each of the eight genomic RNA segments is difficult [22]. 

In the present study, we describe the characterization of a H2N9 isolate from a wild duck in Korea for the first time. The complete genomic sequence of this isolate was obtained using NGS and its genetic/antigenic variation was analyzed.

## 2. Materials and Methods

### 2.1. Sample Collection

A total of 500 fresh fecal droppings of wild birds were sampled and pooled in groups of up to three using sterile swabs in Korea on December 11, 2018. The samples were stored at 2 to 8 °C and shipped to the laboratory within 12 h for further analysis.

### 2.2. Isolation of Influenza Virus from Samples

The fecal samples were resuspended in phosphate buffered saline (PBS) containing antibiotic solution (100 U/µL of penicillin and 100 mg/µL of streptomycin) (Merck, St. Louis, MO, USA) and clarified by centrifugation (3000 rpm for 10 min at 4 °C). Supernatants were filtered through a hydrophilic polyethersulfone membrane (0.45 µm pore size; GVS Syringe) (Novatech, Kingwood, TX, USA) to remove eukaryotic cells and bacteria. Processed samples were inoculated into the allantoic cavities of ten-day-old embryonated chicken eggs and incubated at 37 °C for 2–3 days. Allantoic fluids were harvested and viral titers were determined using hemagglutination assays as previously described [23].

### 2.3. RNA Extraction for Subtyping

Viral RNA was extracted from allantoic fluid (640 hemagglutination units (HAU)/mL) using a total RNA extraction kit (Macherey Nagel, Seoul, Korea) according to the manufacturer’s instructions. Finally, RNA was eluted in 60 μL of RNase-free water, and after addition of 20 U of RNase inhibitor (Sigma-Aldrich, St. Louis, MO, USA), was stored at −80 °C until further use. In the negative control, sterile water was added instead of the specimen.

### 2.4. Subtyping Using PCR

To evaluate influenza growth and determine the subtype, conventional real-time RT-PCR for influenza A virus that amplifies the matrix gene coding sequence was performed using total RNA following the WHO guidelines [24]. Host identification was confirmed using a DNA barcode and a 539 bp region of the mitochondrial gene cytochrome *c* oxidase I (COI) using COI sequences from all 260 bird species as previously described [25]. The host of the new isolate was identified using Barcode of Life Data Systems (BOLD; Biodiversity Institute of Ontario, University of Guelph, Guelph, Ontario, Canada) [26].

### 2.5. Sequencing Using Illumina HiSeq X Method for NGS

NGS was conducted by GnCBio (Daejeon, Korea) using HiSeq X as previously reported [26,27]. Briefly, influenza RNA was evaluated using an Agilent RNA 6000 Pico kit (Agilent, Santa Clara, CA, USA), and the concentration was measured using a spectrophotometer of BioPhotometer^®^ (Eppendorf, Hamburg, Germany). The cDNA library of influenza RNA was generated using QIAseq FX Single Cell RNA Library Kit (QIAGEN, Venlo, Netherlands). cDNA concentration was measured using LightCycle qPCR (Roche, Penzberg, Upper Bavaria, Germany), and library size was checked using Agilent High Sensitivity D5000 ScreenTape System (Santa Clara, CA, USA).

### 2.6. Sequence Analysis

Raw sequence reads were quality-trimmed using Trim Galore! (Babraham Bioinformatics, UK) (q = 20). Influenza and non-influenza viruses were classified by DeconSeq (iden = 60) using a database created by downloading influenza virus sequences from the National Center for Biotechnology Information (NCBI) databank. The homology of the segments 4 (HA), 5 (NA), and 8 (NS1), known to have high variability among influenza virus segments, was examined by Basic Local Alignment Search Tool for nucleotides (BLASTn) in the influenza virus database. Among the results, the most homologous and the most common sequences were selected as the reference of this study. Sequence reads classified as influenza virus were mapped to the selected reference sequence using the gsMapper program (iden = 70, Coverage = 40). Consensus sequences obtained by mapping were corrected for sequence errors using a proofread program and open reading frames (ORF) were predicted using ORFfinder (NCBI) [28].

### 2.7. Phylogenetic Tree Analyses

The closest relatives of the viral genes sequenced in this study were identified using the BLAST function in GenBank^®^ (NCBI). Phylogenetic trees were generated by the neighbor-joining method using MEGA6 (Molecular Evolutionary Genetics Analysis version 6.0, Pennsylvania State University, PA, USA). Bootstrap values were calculated based on 1000 alignment replicates.

## 3. Results

### 3.1. Virus Isolation from Fecal Samples of Migratory Birds

Virus isolation was performed from fresh fecal samples of migratory birds in Korea, and an AIV was confirmed from a wild duck (A/wild duck/Korea/K102/2018 (H2N9)) isolate. This isolate was collected from wild bird feces in the Gyeongbuk area (35° 54’ 34.31’’, 128° 49’ 43.61’’) on December 11, 2018. 

The host was identified as *Anas platyrhynchos, A. platyrhynchos x A. crecca, A. poecilorhyncha,* and *Tadorna tadorna* (Figure S1). Due to the similarity of the genes, the species could not be differentiated among the four birds and thus, the host was described as wild duck for our isolate. The sequence of COI (539 bp) is provided in Table S1.

The H2N9 AIV from the wild duck samples (A/wild duck/Korea/K102/2018 (H2N9)) was isolated by inoculating the virus in embryonated chicken eggs, followed by hemagglutination assays (640 HAU/mL in stock) and subtype PCR. 

The alignment lengths for each small data set were PB2, 2280 nucleotides (nt); PB1, 2278 nt; PA, 2121 nt; HA, 1688 nt; NP, 1482 nt; NA, 1384 nt; M, 980 nt; and NS, 822 nt. 

In the sequence analysis, the number of AIV reads was 2,186,688, which accounted for 10.37% of the raw NGS data. Consensus contigs for eight segments of the AIV were obtained by data assembly and the error correction process. Investigation with ORFfinder (NCBI) confirmed that these contigs all have a complete ORF (Table 1). Detailed NGS analysis is provided in Table S2.

### 3.2. Phylogenic Analysis of the Surface and Internal Genes

Phylogenic analysis for the eight genes of the AIV (A/wild duck/Korea/K102/2018) (H2N9) was performed to assess their genetic relationships with those of domestic poultry and wild birds in Korea and neighboring countries, using data from NCBI. All phylogenic analyses of each gene are shown in the separate supplementary information. The results showed that the surface genes (HA and NA) of our H2N9 strain were found to be widespread in various Eurasian countries, whereas the internal genes were found to be limited in East Asia countries.

The HA gene of this virus was similar to that of the H2N3 strain isolated in Mongolia (2015), whereas the NA gene of our isolate was similar to that of the H10N9 strain isolated in Bangladesh (2010). The viral genes PB2 and PA were 98.9% and 99.49% identical to the PB2 and PA genes of A/duck/Jiangsu/SE0261/2018 (H5N3). 

**Figure 1:**
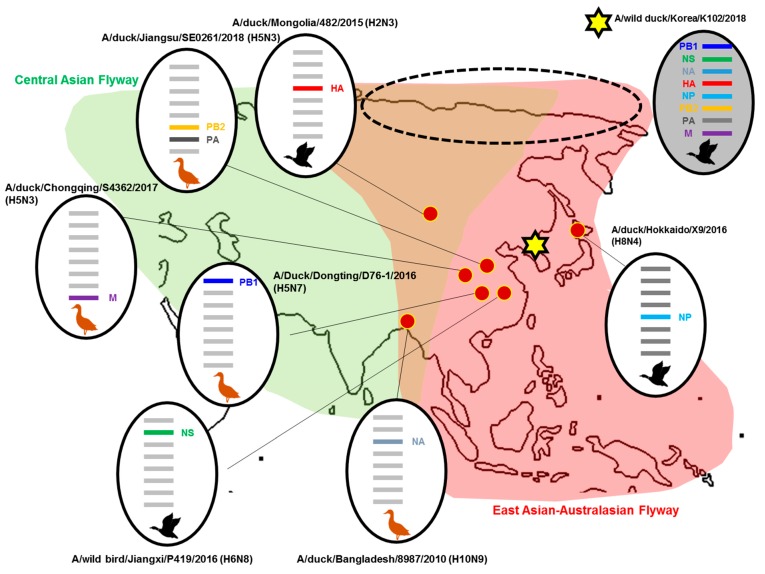
Location of putative origins of genomic compositions of the A/wild duck/Korea/K102/2018 (H2N9). Dotted circles indicate the multiple converging flyways in which reassortments occur.

The remaining genes from our isolate (PB1, NP, M, and NS) showed a close relationship with A/Duck/Dongting/D76-1/2016 (H5N7), A/duck/Hokkaido/X9/2016 (H8N4), A/duck/Chongqing/S4362/2017 (H5N3), and A/wild bird/Jiangxi/P419/2016 (H6N8), respectively. The percent sequence homology for each gene segment from A/wild duck/Korea/K102/2018 (H2N9) compared to the closest genetic relative is shown in Table 2. All strains including the gene related to our strain are shown in Figure 1.

### 3.3. Molecular Characterization of the HA and NA Amino Acid Sequences

The receptor-binding site (RBS) of HA was assessed by analyzing mutations that change host tropism from bird to human (Table 3). The RBS structure is conserved across all HA subtypes and is comprised of the 130 loop (residues 135 to 138), 190 helix (residues 190 to 198), and 220 loop (residues 221 to 228) [29]. In these residues, two adaptive mutations in RBS were necessary to switch the preference from avian- to human-type receptors: E190D and G225D for H1N1, and Q226L and G228S in the case of H2N2 and H3N2 [30]. The HA of our isolate was compared with a strain isolated from a human (A/Korea/426/1968 (H2N2) and three strains isolated from birds (A/northern shoveler/Hong Kong/MPL133/2010, A/northern shoveler/Hong Kong/MPL961/2011, and A/wild waterfowl/Hong Kong/MPL696/2011), which are closely related to the genesis of human H7N9 virus [31].

The HA cleavage site of the A/wild duck/Korea/K102/2018 (H2N9) virus contained the PQIEPR↓GLF (↓ denotes cleavage site) sequence, with a monobasic amino acid (arginine, R) in the HA cleavage site, a signature of LPAI [32]. 

Comparing the receptor-binding sequence of the HA1 gene of our isolate with those of Hong Kong (H2N9) isolates and the human H2N2 isolate, A/Korea/426/1968(H2N2) showed that the amino acid residues at positions 138, 190, 194, and 225 (H3 numbering) are similar. 

However, the amino acid sequence of avian H2N9 isolates, including our strain, is different from that of human isolates at positions 226 and 228 (H3 numbering) because only human isolates (H2N2) exhibit different amino acids in 220 loop (residues 221 to 228) [30]. According to the HA phylogenic tree of H2 subtypes, H2N9 originated in swine and dispersed to two different clades: human and avian.

The NA stalk truncation of 20 amino acids increases virulence in mice and ferrets [33,34,35]. Our isolate showed no truncation in the stalk region of NA residues 69 to 73, similar to the NA genes of H2N9 subtypes isolated in Hong Kong (2010–2011) [33,34,35] (Table 4). 

However, other mutations at positions 26, 107, 144, 146, and 224 in the NA gene, which increases virulence in mice and mammals, were found in our isolate and the reference strains (Table S3). Interestingly, the mutation at position 294, arginine (R) to lysine (K), was only found in A/Shanghai/1/2013 isolated from humans, and this single substitution has been demonstrated to reduce susceptibility to oseltamivir and zanamivir [36].

Shanghai/2013(H7N9) NA gained a substitution of the amino acid at position 294 from R to K among four human H2N9 strains and our isolate showed K. This implies the potential susceptibility of our isolate to anti-influenza drugs. 

### 3.4. Molecular Characterization of the Internal Amino Acid Sequences

Mutation in the ribonucleoprotein complex (PB2, PB1, PA, and NP) could have increased the replication efficiency of the virus in a different host. Mutations in the internal genes were compared with those of H2N9 in wild birds to assess new variations and notably different residues were identified, as shown in Table 4. The mutation E627K that enhances viral replication in mammalian cells in PB2 was not found in our H2N9 isolate [37]. Similar results were observed in other Hong Kong H2N9 isolates, but human H7N9 isolates had this mutation. In addition, the PB2 gene of our H2N9 isolate had several mutations (L89V, K251R, G309D, and H447Q) that may enhance polymerase activity and increase virulence in mice and mammals [39,40,41]. 

The PB1 protein residues D/A3V, V13P, R207K, and S375N/T are known to increase polymerase activity and virulence in mammals [42,43,44,45]. This mutation was found in our H2N9 isolate, Hong Kong avian isolates (H2N9, 2010–2013), and Chinese human isolates (Anhui/1/2013 and Shanghai/1/2013). The PA protein harbored mutations at position 266 (H266R), 277 (F277S), and 515 (S515T) that increase polymerase activity and virulence in mammals and birds [44,46,47].

Two substitutions in the NP protein, V41I and D210E, contribute to viral transmissibility in mammalian cells [48]. Moreover, the A184K substitution in the NP protein increases replication and pathogenicity of H5N1 [49]. All three mutations were found in A/wild duck/Korea/K102/2018 (H2N9). In addition, our H2N9 isolate had signature mutations in other proteins that are associated with the increased virulence factor of the influenza virus A H5N1 strain in mice, such as N30D and T215A in M1 and P42S in NS1 [38,50,51,52].

In addition, similar to the 1918 H1N1 pandemic strain and avian H5N1 strain, our H2N9 isolate had a PDZ domain-binding at the C-terminus of NS1, reflecting a potential adaptation in mice. Mutated residues of NA and six internal genes are summarized in Table 4 and Table S3.

## 4. Discussion

In wild birds, AIVs co-circulate and their reassortments can cause severe outbreaks in poultry and other animals by crossing the species barrier. HPAI evolve from LPAI, and one of most notable viral strains is Chinese H7N9 virus, which evolved from LPAIs (H7N3, H2N11, H2N9, and H9N2) [13]. The other is human H2N2 in 1957, which evolved from avian H2N2 [14]. Therefore, continuous surveillance of LPAIs is necessary.

The peninsular Republic of Korea is crossed by the East Asian Australasian and West Pacific Flyways and is near the Central Asian Flyway. This creates diversity in migratory birds and in AIVs. In our study, phylogenetic analysis demonstrated that the HA and NA genes of A/wild duck/Korea/K102/2018 (H2N9) belonged to the Eurasian lineage, with HA and NA genes showing the closest relationship to A/duck/Mongolia/482/2015 (H2N3) and A/duck/Bangladesh/8987/2010 (H10N9), respectively. In contrast, the internal genes (PB2, PB1, NP, PA, M, and NS) were of East Asian lineage, with the six gene segments of our H2N9 isolate being most closely related to those in other countries such as China (PB1, PA, M, and NS) and Japan (H8N6). Among the internal genes, only the PB1 gene of our H2N9 strain was grouped with Korean AIV strains (A/wild waterfowl/Korea/F14-5/2016(H6N1), A/wild waterfowl/Korea/F7-18/2018 (H4N8), and A/mallard/Korea/F94-16/2017 (H4N6). The remaining genes were absent from the Korea strain groups.

The monobasic cleavage motif PQIEPR↓GLF (underbar, critical basic residue/s; ↓ denotes cleavage site) is typically activated by a trypsin-like serine protease and this requirement restricts the tropism of influenza viruses to the types of tissues that express the proteases for activation, indicating that this isolate is an LPAI with a monobasic cleavage motif [53].

On the contrary, the multi-basic cleavage motif PQGERRRKKR↓G of the HA belongs to the consensus site cleaved by furin, a ubiquitously expressed mammalian enzyme that allows viruses to multiply in mammals rapidly, leading to high pathogenicity of AIV [53,54].

According to HA gene analysis, the motif PQIEPR↓GLF in the cleavage site between HA1 and HA2 of A/wild duck/Korea/K102/2018 (H2N9) was not different from that of an H2N2 isolate from a human sample (A/Korea/426/1968) showing PQIESR↓GLF, a monobasic cleavage site. Human A/Korea/426/1968 may achieve adaptation in humans by substitution of different amino acids in the 220 loop (Table 3). 

In avian H2 influenza viruses, HA receptor-binding-site residues corresponding to the amino acids at positions 138, 190, 194, 225, 226, and 228 (using the H3 numbering system) are highly conserved [28]. Among these sites, the 220 loop (residues 221 to 228) is known to be involved in the increased affinity to the human receptor [29]. In human H2 and H3 viruses, leucine and serine substitutions at positions 226 and 228, respectively, have been shown to accompany their adaptation from avian to human hosts [55]. The substitutions Q226L and G228S were not found in the HA gene of A/wild duck/Korea/K102/2018 (H2N9), which is consistent with H2N9 isolated from Hong Kong, indicating that the newly isolated H2N9 in Korea does not have enhanced human infectivity. In the HA gene of A/Korea/426/1968 isolated from humans, Q226L was not found, whereas position 228 contained a different mutation (228R). Therefore, receptor tropism of the new Korean H2N9 isolate did not change from avian to human.

A deletion in the NA stalk region at position 69–73 that increases virulence in mice was not found in our strain and the Hong Kong strains, whereas this deletion is present in human strains (A/Shanghai/1/2013/H7N9 and A/Anhui/1/2013/H7N9). Interestingly, the mutations M26I, 107V, R144K, and N144S, which may increase virulence in mammals, were found in both our isolate and the reference strain of H2N9. The A/wild duck/Korea/K102/2018 (H2N9) harbored no mutation at position E627K of PB2, which plays an important role in the adaption of AIVs to mammals. These results imply that the avian A/wild duck/Korea/K102/2018 (H2N9) may not exhibit host tropism to humans.

In contrast, other mutations that enhance polymerase activity and increase virulence in mammals were commonly present in both our H2N9 isolate and the references isolates. Consequently, the genotype of the A/wild duck/Korea/K102/2018 (H2N9) strain might be the result of reassortment events that occurred during wild bird migration.

To date, H2N9 has never been discovered in Korea, and to the best of our knowledge, this is the first study to describe a reassortant avian H2N9 virus from wild duck fecal samples. Although H2N9 belongs to LPAI, it is still necessary to monitor its presence in Korea because it is known to be one of the multiple reassortments involved in the induction of HPAI H7N9 from LPAI H7N9 in humans [56].

**Figure 2:**
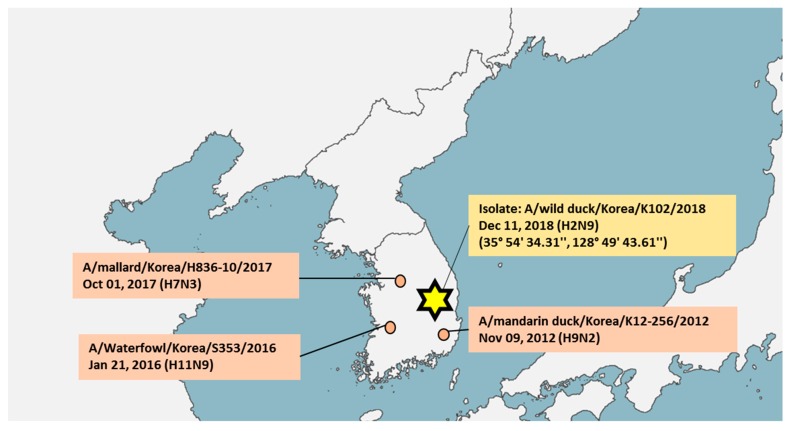
The geographical location of pre-existing subtypes (H7N3, H11N9, and H9N2) and novel isolate (A/wild duck/Korea/K102/2018 (H2N9)) in Korea.

Although there was no outbreak of H7N9 in Korea, the avian LPAI H7N9 virus was introduced into Korea [15,16]. Therefore, our study emphasizes the importance of understanding avian influenza genetic diversity as a H7N9 outbreak may occur in the future. As other subtypes (H7N3 [18], H9N2 [19], and H11N9 [20]) and multiple reassortments of H7N9 HPAI have been co-circulating in Korea in the wild within a distance of 500 km (Figure 2), the discovery of H2N9 in Korea must be recognized as an early warning of the potential for HPAI H7N9 outbreak in the country. As the migratory birds are capable of non-stop flights of up to 5000 km [57], there is potential for multiple reassortment by pre-existing subtypes in Korea. 

There is growing evidence of AIV reassortment at stopover sites, where migratory flyways intersect and thus, the frequency of intercontinental reassortment is likely a reflection of overlapping migratory pathways [58,59,60]. In this context, the reassortment of H2N9 may have occurred in Russian territory as both East Asian-Australasian and Central Asian flyways converge there (dotted line in Figure 1).

In conclusion, LPAI A/wild duck/Korea/K102/2018 (H2N9) is the first reported isolate in Korea and may contain properties that would enhance virulence in the mammalian host. Owing to potential reassortments between H2N9 and other pre-existing viral subtypes in Korea, surveillance and characterization of all these viral subtypes is essential for management of AIV health risk in the early stages. 

Therefore, continuous surveillance and genetic analysis to characterize LPAI viruses, including the H7, H9, H11, and H2 subtypes of AIV are strongly encouraged. 

## Figures and Tables

**Table 1:** Genomic sequence of A/wild duck/Korea/K102/2018 (H2N9) obtained by NGS.

Gene Name	ID	# of Mapped Reads	R_ORF ^a^			S_ORF ^b^		
			Length (bp)	S_Position^c^	E_Position^d^	Length (bp)	S_Position	E_Position
PB2	KF260787	17,148	2280	1	2280	2274	19	2292
PB1	KF260543	17,999	2274	1	2274	2274	3	2276
PA	KF260299	16,372	2151	1	2151	2151	1	2151
HA	KF258945	53,303	1689	1	1689	1689	1	1689
NP	KF259811	97,019	1497	1	1497	1506	1	1506
NA	KF259722	4713	1413	1	1413	1395	5	1399
M	KF259292	333,564	759	1	759	759	1	759
NS1	KF260055	35,329	693	1	693	690	2	691

^a^ R_ORF, ORF of the reference sequence; ^b^ S_ORF, ORF of the sample sequence; ^c^ S_position, start position at ORF of consensus sequence; ^d^ E_position, end position at ORF of consensus sequence.

**Table 2:** Sequence homology of the whole genome of the A/wild duck/Korea/K102/2018 (H2N9) avian influenza virus (AIV) compared to influenza sequences available in NCBI.

Gene	A/Wild Duck/Korea/K102/2018 (H2N9) (GenBank Accession #)	Highest Percentage of Nucleotide Identity (GenBank Accession #)	% Nucleotide Identity ^a^
PB2	MN511808	A/duck/Jiangsu/SE0261/2018 (H5N3) (MN171447.1)	98.90%
PB1	MN511809	A/Duck/Dongting/D76-1/2016 (H5N7) (MF362101.1)	98.46%
PA	MN511810	A/duck/Jiangsu/SE0261/2018 (H5N3) (MN171449.1)	99.49%
HA	MN511811	A/duck/Mongolia/482/2015 (H2N3) (LC121372.1)	98.34%
NP	MN511812	A/duck/Hokkaido/X9/2016 (H8N4) (MK978905.1)	99.39%
NA	MN511813	A/duck/Bangladesh/8987/2010 (H10N9) (MH071484.1)	96.45%
M	MN511814	A/duck/Chongqing/S4362/2017 (H5N3) (MN171429.1)	99.07%
NS	MN511815	A/wild bird/Jiangxi/P419/2016 (H6N8) (KX867861.1)	99.25%

^a^ Representative viruses with the highest nucleotide sequence identity found in the NCBI database in August, 2019, are listed.

**Table 3:** Comparison of amino acid sequence in HA receptor-binding sites of human and avian H2 influenza virus isolates.

Strains	HA Receptor-Binding Residues (H3 Numbering)						
	138	190	194	225	226	228	Cleavage Site
K/2018 ^a^	A	E	L	G	Q	G	PQIEPR↓GLF
H/MPL133 ^b^	A	E	L	G	Q	G	PQIESR↓GLF
H/MPL961 ^c^	A	E	L	G	Q	G	PQIESR↓GLF
H/MPL696 ^d^	A	E	L	G	Q	G	PQIESR↓GLF
K/426 ^e^	A	E	L	G	L	R	PQIESR↓GLF

^a^ K/2018, A/wild duck/Korea/K102/2018 (H2N9); ^b^ H/MPL133, A/northern shoveler/Hong Kong/MPL133/2010 (H2N9); ^c^ H/MPL961, A/northern shoveler/Hong Kong/MPL961/2011(H2N9); ^d^ H/MPL696, A/wild waterfowl/Hong Kong/MPL696/2011(H2N9); ^e^ K/426, A/Korea/426/1968(H2N2).

**Table 4:** Genetic analysis of the NA and internal gene of H2N9.

Virus Strain	NA		PB2	NS1
	Truncation at 69–73 aa	R294K	E627K	P42S
**K/2018 ^a^**	QISNT	R	E	No deletion
**S/2013 ^b^**	Deletion	K	K	Deletion
**A/2013 ^c^**	Deletion	R	K	Deletion
**H/MPL13 ^d^**	QISNT	R	E	Deletion
**H/MPL961 ^e^**	QISNT	R	E	No deletion
Comments	Deletion of amino acids 69–73: Increased virulence in mice [33]	Reduced susceptibility to oseltamivir and zanamivir [36]	Mammalian host adaptation [37]	Lack of PDZ domain binding motif: Decreased virulence in mice [38]

^a^ K/2018, A/wild duck/Korea/K102/2018 (H2N9); ^b^ S/2013, A/Shanghai/1/2013 (H7N9-human isolate); ^c^ A/2013, A/Anhui/1/2013 (H7N9-human isolate); ^d^ H/MPL133, A/northern shoveler/Hong Kong/MPL133/2010 (H2N9); ^e^ H/MPL961, A/northern shoveler/Hong Kong/MPL961/2011(H2N9).

## Supplementary Materials

The following are available online at https://www.mdpi.com/1999-4915/11/11/1046/s1, Figure S1: Information of bird species identification, Table S1: Initial input of barcoding, Table S2: Detailed NGS analysis, Table S3: Amino acid residues of the NA, PB2, PB1, PA, NP, NS1 and M1 gene of A/wild duck/ Korea/K102/2018 (H2N9).

## References

[1] Alexander D.J. (2007). An overview of the epidemiology of avian influenza. Vaccine.

[2] Influenza Type A Viruses. https://www.cdc.gov/flu/avianflu/influenza-a-virus-subtypes.htm.

[3] Senne D., Panigrahy B., Kawaoka Y., Pearson J., Süss J., Lipkind M., Kida H., Webster R. (1996). Survey of the hemagglutinin (HA) cleavage site sequence of H5 and H7 avian influenza viruses: Amino acid sequence at the HA cleavage site as a marker of pathogenicity potential. Avian Dis..

[4] de Graaf M., Fouchier R.A. (2014). Role of receptor binding specificity in influenza A virus transmission and pathogenesis. EMBO J..

[5] Ha Y., Stevens D.J., Skehel J.J., Wiley D.C. (2001). X-ray structures of H5 avian and H9 swine influenza virus hemagglutinins bound to avian and human receptor analogs. Proc. Natl. Acad. Sci. USA.

[6] Verhagen J.H., Lexmond P., Vuong O., Schutten M., Guldemeester J., Osterhaus A.D., Elbers A.R., Slaterus R., Hornman M., Koch G. (2017). Discordant detection of avian influenza virus subtypes in time and space between poultry and wild birds; Towards improvement of surveillance programs. PLoS ONE.

[7] Kishida N., Sakoda Y., Shiromoto M., Bai G.R., Isoda N., Takada A., Laver G., Kida H. (2008). H2N5 influenza virus isolates from terns in Australia: Genetic reassortants between those of the Eurasian and American lineages. Virus Genes.

[8] Ma W., Vincent A.L., Gramer M.R., Brockwell C.B., Lager K.M., Janke B.H., Gauger P.C., Patnayak D.P., Webby R.J., Richt J.A. (2007). Identification of H2N3 influenza A viruses from swine in the United States. Proc. Natl. Acad. Sci. USA.

[9] Joseph U., Linster M., Suzuki Y., Krauss S., Halpin R.A., Vijaykrishna D., Fabrizio T.P., Bestebroer T.M., Maurer-Stroh S., Webby R.J. (2015). Adaptation of pandemic H2N2 influenza A viruses in humans. J. Virol..

[10] Beaudoin A., Gramer M., Gray G.C., Capuano A., Setterquist S., Bender J. (2010). Serologic survey of swine workers for exposure to H2N3 swine influenza A. Influ. Other Respir. Viruses.

[11] Killian M.L., Zhang Y., Panigrahy B., Trampel D., Yoon K.J. (2011). Identification and characterization of H2N3 avian influenza virus from backyard poultry and comparison with novel H2N3 swine influenza virus. Avian Dis..

[12] Jonassen C.M., Handeland K. (2007). Avian influenza virus screening in wild waterfowl in Norway, 2005. Avian Dis..

[13] Gao R., Cao B., Hu Y., Feng Z., Wang D., Hu W., Chen J., Jie Z., Qiu H., Xu K. (2013). Human infection with a novel avian-origin influenza A (H7N9) virus. N. Engl. J. Med..

[14] Linster M., Schrauwen E.J.A., van der Vliet S., Burke D.F., Lexmond P., Bestebroer T.M., Smith D.J., Herfst S., Koel B.F., Fouchier R.A.M. (2019). The Molecular Basis for Antigenic Drift of Human A/H2N2 Influenza Viruses. J. Virol..

[15] Lee Y.N., Cheon S.H., Lee E.K., Heo G.B., Bae Y.C., Joh S.J., Lee M.H., Lee Y.J. (2018). Pathogenesis and genetic characteristics of novel reassortant low-pathogenic avian influenza H7 viruses isolated from migratory birds in the Republic of Korea in the winter of 2016–2017. Emerg. Microbes Infect..

[16] Kang H.M., Park H.Y., Lee K.J., Choi J.G., Lee E.K., Song B.M., Lee H.S., Lee Y.J. (2014). Characterization of H7 influenza A virus in wild and domestic birds in Korea. PLoS ONE.

[17] Chinese-origin H7N9 Avian Influenza Spread in Poultry and Human Exposure. http://www.fao.org/3/CA3206EN/ca3206en.pdf.

[18] Kim H.R., Park C.K., Lee Y.J., Oem J.K., Kang H.M., Choi J.G., Lee O.S., Bae Y.C. (2012). Low pathogenic H7 subtype avian influenza viruses isolated from domestic ducks in South Korea and the close association with isolates of wild birds. J. Gen. Virol..

[19] Kang M., Jang H.K. (2017). Genetics and biological property analysis of Korea lineage of influenza A H9N2 viruses. Vet. Microbiol..

[20] Le T.B., Lee I.H., Kim H.S., Oh S.K., Seo S.H. (2017). Genetic analysis of a novel reassortant H11N9 Isolated from waterfowl in South Korea in 2016. Virus Genes.

[21] Van den Hoecke S., Verhelst J., Vuylsteke M., Saelens X. (2015). Analysis of the genetic diversity of influenza A viruses using next-generation DNA sequencing. BMC Genom..

[22] Croville G., Soubies S.M., Barbieri J., Klopp C., Mariette J., Bouchez O., Camus-Bouclainville C., Guerin J.L. (2012). Field monitoring of avian influenza viruses: Whole-genome sequencing and tracking of neuraminidase evolution using 454 pyrosequencing. J. Clin. Microbiol..

[23] OIE—World Organisation for Animal Health; Paris, France, 2019. http://www.fao.org/3/CA3206EN/ca3206en.pdf.

[24] World Health Organization; Geneva, Switzerland, 2017. https://www.who.int/influenza/gisrs_laboratory/WHO_information_for_the_molecular_detection_of_influenza_viruses_20171023_Final.pdf.

[25] Hebert P.D., Stoeckle M.Y., Zemlak T.S., Francis C.M. (2004). Identification of Birds through DNA Barcodes. PLoS Biol..

[26] Lee D.H., Lee H.J., Lee Y.J., Kang H.M., Jeong O.M., Kim M.C., Kwon J.S., Kwon J.H., Kim C.B., Lee J.B. (2010). DNA barcoding techniques for avian influenza virus surveillance in migratory bird habitats. J. Wildl. Dis..

[27] Ambardar S., Gupta R., Trakroo D., Lal R., Vakhlu J. (2016). High Throughput Sequencing: An Overview of Sequencing Chemistry. Indian J. Microbiol..

[28] Hackl T., Hedrich R., Schultz J., Forster F. (2014). proovread: Large-scale high-accuracy PacBio correction through iterative short read consensus. Bioinformatics.

[29] Skehel J.J., Wiley D.C. (2000). Receptor binding and membrane fusion in virus entry: The influenza hemagglutinin. Annu. Rev. Biochem..

[30] Lazniewski M., Dawson W.K., Szczepinska T., Plewczynski D. (2018). The structural variability of the influenza A hemagglutinin receptor-binding site. Brief. Funct. Genom..

[31] Lam T.T.-Y., Wang J., Shen Y., Zhou B., Duan L., Cheung C.-L., Ma C., Lycett S.J., Leung C.Y.-H., Chen X. (2013). The genesis and source of the H7N9 influenza viruses causing human infections in China. Nature.

[32] Nao N., Yamagishi J., Miyamoto H., Igarashi M., Manzoor R., Ohnuma A., Tsuda Y., Furuyama W., Shigeno A., Kajihara M. (2017). Genetic Predisposition To Acquire a Polybasic Cleavage Site for Highly Pathogenic Avian Influenza Virus Hemagglutinin. mBio.

[33] Bi Y., Xiao H., Chen Q., Wu Y., Fu L., Quan C., Wong G., Liu J., Haywood J., Liu Y. (2016). Changes in the Length of the Neuraminidase Stalk Region Impact H7N9 Virulence in Mice. J. Virol..

[34] Blumenkrantz D., Roberts K.L., Shelton H., Lycett S., Barclay W.S. (2013). The short stalk length of highly pathogenic avian influenza H5N1 virus neuraminidase limits transmission of pandemic H1N1 virus in ferrets. J. Virol..

[35] Matsuoka Y., Swayne D.E., Thomas C., Rameix-Welti M.A., Naffakh N., Warnes C., Altholtz M., Donis R., Subbarao K. (2009). Neuraminidase stalk length and additional glycosylation of the hemagglutinin influence the virulence of influenza H5N1 viruses for mice. J. Virol..

[36] Hai R., Schmolke M., Leyva-Grado V.H., Thangavel R.R., Margine I., Jaffe E.L., Krammer F., Solórzano A., García-Sastre A., Palese P. (2013). Influenza A(H7N9) virus gains neuraminidase inhibitor resistance without loss of in vivo virulence or transmissibility. Nat. Commun..

[37] Yamayoshi S., Kiso M., Yasuhara A., Ito M., Shu Y., Kawaoka Y. (2018). Enhanced Replication of Highly Pathogenic Influenza A(H7N9) Virus in Humans. Emerg. Infect. Dis..

[38] Kageyama T., Fujisaki S., Takashita E., Xu H., Yamada S., Uchida Y., Neumann G., Saito T., Kawaoka Y., Tashiro M. (2013). Rapid communication Genetic analysis of novel avian A (H7N9) influenza viruses isolated from patients in China, February to April 2013. Euro Surveill..

[39] Husain M. (2014). Avian influenza A (H7N9) virus infection in humans: Epidemiology, evolution, and pathogenesis. Infect. Genet. Evol..

[40] Katz J.M., Lu X., Tumpey T.M., Smith C.B., Shaw M.W., Subbarao K. (2000). Molecular correlates of influenza A H5N1 virus pathogenesis in mice. J. Virol..

[41] Prokopyeva E., Sobolev I., Prokopyev M., Shestopalov A. (2016). Adaptation of influenza A (H1N1) pdm09 virus in experimental mouse models. Infect. Genet. Evol..

[42] Govorkova E.A., Rehg J.E., Krauss S., Yen H.-L., Guan Y., Peiris M., Nguyen T.D., Hanh T.H., Puthavathana P., Long H.T. (2005). Lethality to ferrets of H5N1 influenza viruses isolated from humans and poultry in 2004. J. Virol..

[43] Gabriel G., Dauber B., Wolff T., Planz O., Klenk H.-D., Stech J. (2005). The viral polymerase mediates adaptation of an avian influenza virus to a mammalian host. Proc. Natl. Acad. Sci. USA.

[44] Hulse-Post D., Franks J., Boyd K., Salomon R., Hoffmann E., Yen H., Webby R., Walker D., Nguyen T., Webster R. (2007). Molecular changes in the polymerase genes (PA and PB1) associated with high pathogenicity of H5N1 influenza virus in mallard ducks. J. Virol..

[45] Taubenberger J.K., Reid A.H., Lourens R.M., Wang R., Jin G., Fanning T.G. (2005). Characterization of the 1918 influenza virus polymerase genes. Nature.

[46] Leung B.W., Chen H., Brownlee G.G. (2010). Correlation between polymerase activity and pathogenicity in two duck H5N1 influenza viruses suggests that the polymerase contributes to pathogenicity. Virology.

[47] Mei K., Liu G., Chen Z., Gao Z., Zhao L., Jin T., Yu X., Chen Q. (2016). Deep sequencing reveals the viral adaptation process of environment-derived H10N8 in mice. Infect. Genet. Evol..

[48] Zhu W., Zou X., Zhou J., Tang J., Shu Y. (2015). Residues 41V and/or 210D in the NP protein enhance polymerase activities and potential replication of novel influenza (H7N9) viruses at low temperature. Virol. J..

[49] Wasilenko J.L., Sarmento L., Pantin-Jackwood M.J. (2009). A single substitution in amino acid 184 of the NP protein alters the replication and pathogenicity of H5N1 avian influenza viruses in chickens. Arch. Virol..

[50] Lycett S., Ward M., Lewis F., Poon A., Pond S.K., Brown A.L. (2009). Detection of mammalian virulence determinants in highly pathogenic avian influenza H5N1 viruses: Multivariate analysis of published data. J. Virol..

[51] Jiao P., Tian G., Li Y., Deng G., Jiang Y., Liu C., Liu W., Bu Z., Kawaoka Y., Chen H. (2008). A single-amino-acid substitution in the NS1 protein changes the pathogenicity of H5N1 avian influenza viruses in mice. J. Virol..

[52] Fan S., Deng G., Song J., Tian G., Suo Y., Jiang Y., Guan Y., Bu Z., Kawaoka Y., Chen H. (2009). Two amino acid residues in the matrix protein M1 contribute to the virulence difference of H5N1 avian influenza viruses in mice. Virology.

[53] Tse L.V., Hamilton A.M., Friling T., Whittaker G.R. (2014). A novel activation mechanism of avian influenza virus H9N2 by furin. J. Virol..

[54] El-Shesheny R., Kandeil A., Bagato O., Maatouq A.M., Moatasim Y., Rubrum A., Song M.S., Webby R.J., Ali M.A., Kayali G. (2014). Molecular characterization of avian influenza H5N1 virus in Egypt and the emergence of a novel endemic subclade. J. Gen. Virol..

[55] Connor R.J., Kawaoka Y., Webster R.G., Paulson J.C. (1994). Receptor specificity in human, avian, and equine H2 and H3 influenza virus isolates. Virology.

[56] Watanabe T., Watanabe S., Maher E.A., Neumann G., Kawaoka Y. (2014). Pandemic potential of avian influenza A (H7N9) viruses. Trends Microbiol..

[57] Hedenström A. (2010). Extreme endurance migration: What is the limit to non-stopflight?. PLoS Biol..

[58] Ramey A.M., Pearce J.M., Ely C.R., Guy L.M., Irons D.B., Derksen D.V., Ip H.S. (2010). Transmission and reassortment of avian influenza viruses at the Asian-North American interface. Virology.

[59] Li Y., Shi J., Zhong G., Deng G., Tian G., Ge J., Zeng X., Song J., Zhao D., Liu L. (2010). Continued evolution of H5N1 influenza viruses in wild birds, domestic poultry, and humans in China from 2004 to 2009. J Virol..

[60] Pearce J.M., Ramey A.M., Flint P.L., Koehler A.V., Fleskes J.P., Franson J.C., Hall J.S., Derksen D.V., Ip H.S. (2009). Avian influenza at both ends of a migratory flyway: Characterizing viral genomic diversity to optimize surveillance plans for North America. Evol. Appl..

