# Supplementary material for: Molecular Characterization of a Novel Avian Influenza A (H2N9) Strain Isolated from Wild Duck in Korea in 2018
Source: Viruses. 2019 Nov 10;11(11):1046. doi: 10.3390/v11111046 (PMC6893532; doi:10.3390/v11111046)
Supplement: Supplementary file 1 [file viruses-11-01046-s001.pdf]

Supplementary information

Molecular characterization of a novel avian influenza A (H2N9) strain isolated from wild duck in Korea in 2018

Seon-Ju Yeo*1, Duc Duong Than*1, Hong-Seog Park2, Haan Woo Sung§3, Hyun Park§1

Fig. S1. Information of bird species identification

Table S1. Initial input of barcoding

Table S2. Detailed NGS analysis

Table S3. Amino acid residues of the NA, PB2, PB1, PA, NP, NS1 and M1 gene of A/wild duck/ Korea/K102/2018 (H2N9)

Reference

All phylogenetic trees.(HA, M, NA, NP, NS, PA, PB1, PB2)

BOLD TaxonID Taxonomy Report

Order	Family	Species
Anseriformes[99 individuals][4 species]		
	Anatidae[99 individuals][4 species]	
		Anas platyrhynchos[81 individuals]
		Anas platyrhynchos x A. crecca[1 individuals]
		Anas poecilorhyncha[16 individuals]
		Tadorna tadorna[1 individuals]

Fig. S1. Information of bird species identification

Table S1. Initial input of barcoding

AACGTGATCGTCACCGCTCACGCCTTCGTAATAATCTTCTTCATGGTAATGCCCATCATAATTGGAGGGTTCGGCAACTGATTGGTCCCCCTGATAATCGGTGCCCCCGACATAGCATTCCCACGAATAAACAACATAAGCTTCT
GACTCCTCCCACCATCATTCCTCCTTCTACTCGCCTCATCCACTGTAGAAGCTGGCGCTGGTACGGGTTGAACCGTATACCCACCTCTAGCAGGCAACCTAGCCCACGCCGGAGCCTCAGAGGACCTGGCTATCTTCTCACTTC
ACCTGGCTGGTGTCTCCTCCATCCTCGGAGCCATTAACTTCATTACCACAGCCATCAACATAAAACCCCCCGCACTCTCACAATACCAAACCCCACTTTTCGTCTGATCAGTCCTAATTACCGCCATCCTGCTCCTCCTATCACT
CCCCGTCCTCGCCGCCGGCATCACAATGCTACTAACCGACCGAAACCTAAACACCACATTCTTTGATCCTGCCGGAGGGCGAGACACAATCCTGTACCAACACCT

Table S2. Detailed NGS analysis

	Reference						Sequenced Sample								R_ORF			S_ORF					
**Sample**	**Segment#**	**gene name**	**desc.**	**Accession_ID**	**desc. all**	**ref bp**	**# of Pre-processed reads**	**# of Influenza Virus extracted reads**	**# of non-Influenza Virus reads**	**Virus reads %**	**#M_Reads**	**Unique Matches**	**S_Con._bp**	**%Cov.(S/R)**	**Length**	**S_position**	**E_position**	**Length**	**S_position**	**E_position**	**method**	**R_ORF - S_ORF length**	**Contig Count**
K102	1	PB2	A/duck/Guangdong/707/2000/H2N9	KF260787	gi|523793430|gb|KF260787|Influenza A virus (A/duck/Guangdong/707/2000(H2N9)) segment 1 polymerase PB2 (PB2) gene, complete cds	2280	23263654	2186688	21076966	0.103748	17148	0.029	2292	1	2280	1	2280	2274	19	2292	standard :segment 8	6	1
	2	PB1, PB1-F2		KF260543	gi|523792777|gb|KF260543|Influenza A virus (A/duck/Guangdong/707/2000(H2N9)) segment 2 polymerase PB1 (PB1) and PB1-F2 protein (PB1-F2) genes, complete cds	2277					17999	0.03	2280	1	2274	1	2274	2274	3	2276		0	1
	3	PA, PA-X		KF260299	gi|523792051|gb|KF260299|Influenza A virus (A/duck/Guangdong/707/2000(H2N9)) segment 3 polymerase PA (PA) and PA-X protein (PA-X) genes, complete cds	2151					16372	0.027	2151	1	2151	1	2151	2151	1	2151		0	1
	4	HA		KF258945	gi|523788781|gb|KF258945|Influenza A virus (A/duck/Guangdong/707/2000(H2N9)) segment 4 hemagglutinin (HA) gene, complete cds	1689					53303	0.089	1688	1	1689	1	1689	1689	1	1689		0	1
	5	NP		KF259811	gi|523790757|gb|KF259811|Influenza A virus (A/duck/Guangdong/707/2000(H2N9)) segment 5 nucleocapsid protein (NP) gene, complete cds	1497					97019	0.162	1505	0.99	1497	1	1497	1506	1	1506		-9	1
	6	NA		KF259722	gi|523790579|gb|KF259722|Influenza A virus (A/duck/Guangdong/707/2000(H2N9)) segment 6 neuraminidase (NA) gene, complete cds	1413					4713	0.008	1419	0.9795	1413	1	1413	1395	5	1399		18	1
	7	M2, M1		KF259292	gi|523789549|gb|KF259292|Influenza A virus (A/duck/Guangdong/707/2000(H2N9)) segment 7 matrix protein 2 (M2) and matrix protein 1 (M1) genes, complete cds	982					333564	0.556	980	0.999	759	1	759	759	1	759		0	1
	8	NEP, NS1		KF260055	gi|523791319|gb|KF260055|Influenza A virus (A/duck/Guangdong/707/2000(H2N9)) segment 8 nuclear export protein (NEP) and nonstructural protein 1 (NS1) genes, complete cds	845					35329	0.059	822	0.9728	693	1	693	690	2	691		3	1

Table S3. Amino acid residues of the NA, PB2, PB1, PA, NP, NS1 and M1 gene of A/wild duck/ Korea/K102/2018 (H2N9)

Gene segments	Amino acid	K/2018[a]	S/2013[b]	A/2013[c]	H/MPL133[d]	H/MPL961[e]	Comments	Reference
NA	M26I	I	I	I	I	I	M26I: Increased virulence in mice	[1]
	I107V	V	V	V	V	V	I107V: Increased virulence in mice	[2]
	R144K	K	K	K	K	K	R144K: Increased virulence in mice and mammals	[3]
	N146S	S	S	S	S	S	N147S: Increased virulence in mammals	[1]
	T224I	I	I	I	I	I	T224I: Increased virulence in mammals	[4-5]
PB2	L89V	V	V	V	V	V	L89V: Enhanced polymerase activity. Increased virulence in mice	[6-7]
	K251R	R	R	R	R	R	K251R: Increased virulence in mice	[8]
	G309D	D	D	D	D	D	G309D: Enhanced polymerase activity. Increased virulence in mice	[7]
	H447Q	Q	Q	Q	Q	Q	H447Q: Increased polymerase activity. Increased virulence in mammals.	[9-10]
PB1	D/A3V	V	V	V	V	V	D/A3V: Increased polymerase activity. Increased virulence in mammals.	[9-10]
	V13P	P	P	P	P	P	V13P: Increased polymerase activity. Increased virulence in mammals. Mammalian host marker	[11-12]
	R207K	K	K	K	K	K	R207K: Increased polymerase activity in mammalian cells	[13]
	S375N/T	N	N	N	N	N	S375N/T: Increased polymerase activity. Increased virulence in mammals. Human host marker	[9-10, 14]
PA	H266R	R	R	R	R	R	H266R: Increased polymerase activity. Increased virulence in mammals and birds	[15]
	F277S	S	S	S	S	S	F277S: contributed to the virulence and mammalian adaptation	[16]
	S515T	T	T	T	T	T	S515T: Increased polymerase activity. Increased virulence in mammals and birds	[13, 15]

NP	V41I	I	I	I	I	I	V41I: Contribute to viral transmissibility	[17]
	A184K	K	K	K	K	K	A184K: Increased the replication and pathogenicity of H5N1	[18-19]
	D210E	E	E	E	E	E	D210E: Contribute to viral transmissibility	[17]
NS1	P42S	S	S	S	S	S	P42S: Increased virulence in mice	[20-21]
M1	N30D	D	D	D	D	D	N30D: Increased virulence in mice	[22-23]
	T215A	A	A	A	A	A	T215A: Increased virulence in mice	[22-23]

[a]K/2018, A/wild duck/Korea/K102/2018 (H2N9);

[b]S/2013, A/Shanghai/1/2013 (H7N9-human isolate)

[c]A/2013, A/Anhui/1/2013 (H7N9-human isolate)

[d]H/MPL133, A/northern shoveler/Hong Kong/MPL133/2010 (H2N9);

[e]H/MPL961, A/northern shoveler/Hong Kong/MPL961/2011(H2N9);

Reference

1. Mok, C. K. P.; Lee, H. H. Y.; Lestra, M.; Nicholls, J. M.; Chan, M. C. W.; Sia, S. F.; Zhu, H.; Poon, L. L. M.; Guan, Y.; Peiris, J. S. M., Amino acid substitutions in polymerase basic protein 2 gene contribute to the pathogenicity of the novel A/H7N9 influenza virus in mammalian hosts. Journal of virology 2014, 88 (6), 3568-3576.

2. Yen, H.-L.; Aldridge, J. R.; Boon, A. C.; Ilyushina, N. A.; Salomon, R.; Hulse-Post, D. J.; Marjuki, H.; Franks, J.; Boltz, D. A.; Bush, D., Changes in H5N1 influenza virus hemagglutinin receptor binding domain affect systemic spread. Proceedings of the National Academy of Sciences 2009, 106 (1), 286-291.

3. Mishin, V. P.; Sleeman, K.; Levine, M.; Carney, P. J.; Stevens, J.; Gubareva, L. V., The effect of the MDCK cell selected neuraminidase D151G mutation on the drug susceptibility assessment of influenza A (H3N2) viruses. Antiviral research 2014, 101, 93-96.

4. Lee, M.; Deng, M.; Lin, Y.; Chang, C.; Shieh, H. K.; Shiau, J.; Huang, C., Characterization of an H5N1 avian influenza virus from Taiwan. Veterinary microbiology 2007, 124 (3-4), 193-201.

5. Katz, J. M.; Lu, X.; Tumpey, T. M.; Smith, C. B.; Shaw, M. W.; Subbarao, K., Molecular correlates of influenza A H5N1 virus pathogenesis in mice. Journal of virology 2000, 74 (22), 10807-10810.

6. To, K. K.-W.; Song, W.; Lau, S.-Y.; Que, T.-L.; Lung, D. C.; Hung, I. F.-N.; Chen, H.; Yuen, K.-Y., Unique reassortant of influenza A (H7N9) virus associated with severe disease emerging in Hong Kong. Journal of Infection 2014, 69 (1), 60-68.

7. Li, J.; Ishaq, M.; Prudence, M.; Xi, X.; Hu, T.; Liu, Q.; Guo, D., Single mutation at the amino acid position 627 of PB2 that leads to increased virulence of an H5N1 avian influenza virus during adaptation in mice can be compensated by multiple mutations at other sites of PB2. Virus research 2009, 144 (1-2), 123-129.

8. Prokopyeva, E.; Sobolev, I.; Prokopyev, M.; Shestopalov, A., Adaptation of influenza A (H1N1) pdm09 virus in experimental mouse models. Infection, genetics and evolution 2016, 39, 265-271.

9. Salomon, R.; Franks, J.; Govorkova, E. A.; Ilyushina, N. A.; Yen, H.-L.; Hulse-Post, D. J.; Humberd, J.; Trichet, M.; Rehg, J. E.; Webby, R. J., The polymerase complex genes contribute to the high virulence of the human H5N1 influenza virus isolate A/Vietnam/1203/04. Journal of Experimental Medicine 2006, 203 (3), 689-697.

10. Govorkova, E. A.; Rehg, J. E.; Krauss, S.; Yen, H.-L.; Guan, Y.; Peiris, M.; Nguyen, T. D.; Hanh, T. H.; Puthavathana, P.; Long, H. T., Lethality to ferrets of H5N1 influenza viruses isolated from humans and poultry in 2004. Journal of virology 2005, 79 (4), 2191-2198.

11. Gabriel, G.; Dauber, B.; Wolff, T.; Planz, O.; Klenk, H.-D.; Stech, J., The viral polymerase mediates adaptation of an avian influenza virus to a mammalian host. Proceedings of the National Academy of Sciences 2005, 102 (51), 18590-18595.

12. Gabriel, G.; Herwig, A.; Klenk, H.-D., Interaction of polymerase subunit PB2 and NP with importin α1 is a determinant of host range of influenza A virus. PLoS pathogens 2008, 4 (2), e11.

13. Hulse-Post, D.; Franks, J.; Boyd, K.; Salomon, R.; Hoffmann, E.; Yen, H.; Webby, R.; Walker, D.; Nguyen, T.; Webster, R., Molecular changes in the polymerase genes (PA and PB1) associated with high pathogenicity of H5N1 influenza virus in mallard ducks. Journal of virology 2007, 81 (16), 8515-8524.

14. Taubenberger, J. K.; Reid, A. H.; Lourens, R. M.; Wang, R.; Jin, G.; Fanning, T. G., Characterization of the 1918 influenza virus polymerase genes. Nature 2005, 437 (7060), 889.

15. Leung, B. W.; Chen, H.; Brownlee, G. G., Correlation between polymerase activity and pathogenicity in two duck H5N1 influenza viruses suggests that the polymerase contributes to pathogenicity. Virology 2010, 401 (1), 96-106.

16. Mei, K.; Liu, G.; Chen, Z.; Gao, Z.; Zhao, L.; Jin, T.; Yu, X.; Chen, Q., Deep sequencing reveals the viral adaptation process of environment-derived H10N8 in mice. Infection, Genetics and Evolution 2016, 37, 8-13.

17. Zhu, W.; Zou, X.; Zhou, J.; Tang, J.; Shu, Y., Residues 41V and/or 210D in the NP protein enhance polymerase activities and potential replication of novel influenza (H7N9) viruses at low temperature. Virology journal 2015, 12 (1), 71.

18. Wasilenko, J. L.; Sarmento, L.; Pantin-Jackwood, M. J., A single substitution in amino acid 184 of the NP protein alters the replication and pathogenicity of H5N1 avian influenza viruses in chickens. Archives of virology 2009, 154 (6), 969-979.

19. El-Shesheny, R.; Kandeil, A.; Bagato, O.; Maatouq, A. M.; Moatasim, Y.; Rubrum, A.; Song, M. S.; Webby, R. J.; Ali, M. A.; Kayali, G., Molecular characterization of avian influenza H5N1 virus in Egypt and the emergence of a novel endemic subclade. J Gen Virol 2014, 95 (Pt 7), 1444-63.

20. Lycett, S.; Ward, M.; Lewis, F.; Poon, A.; Pond, S. K.; Brown, A. L., Detection of mammalian virulence determinants in highly pathogenic avian influenza H5N1 viruses: multivariate analysis of published data. Journal of virology 2009, 83 (19), 9901-9910.

21. Jiao, P.; Tian, G.; Li, Y.; Deng, G.; Jiang, Y.; Liu, C.; Liu, W.; Bu, Z.; Kawaoka, Y.; Chen, H., A single-amino-acid substitution in the NS1 protein changes the pathogenicity of H5N1 avian influenza viruses in mice. Journal of virology 2008, 82 (3), 1146-1154.

22. Fan, S.; Deng, G.; Song, J.; Tian, G.; Suo, Y.; Jiang, Y.; Guan, Y.; Bu, Z.; Kawaoka, Y.; Chen, H., Two amino acid residues in the matrix protein M1 contribute to the virulence difference of H5N1 avian influenza viruses in mice. Virology 2009, 384 (1), 28-32.

23. Kageyama, T.; Fujisaki, S.; Takashita, E.; Xu, H.; Yamada, S.; Uchida, Y.; Neumann, G.; Saito, T.; Kawaoka, Y.; Tashiro, M., Rapid communication Genetic analysis of novel avian A (H7N9) influenza viruses isolated from patients in China, February to April 2013. Euro surveillance: bulletin Europeen sur les maladies transmissibles= European communicable disease bulletin 2013, 18 (15), 20453.

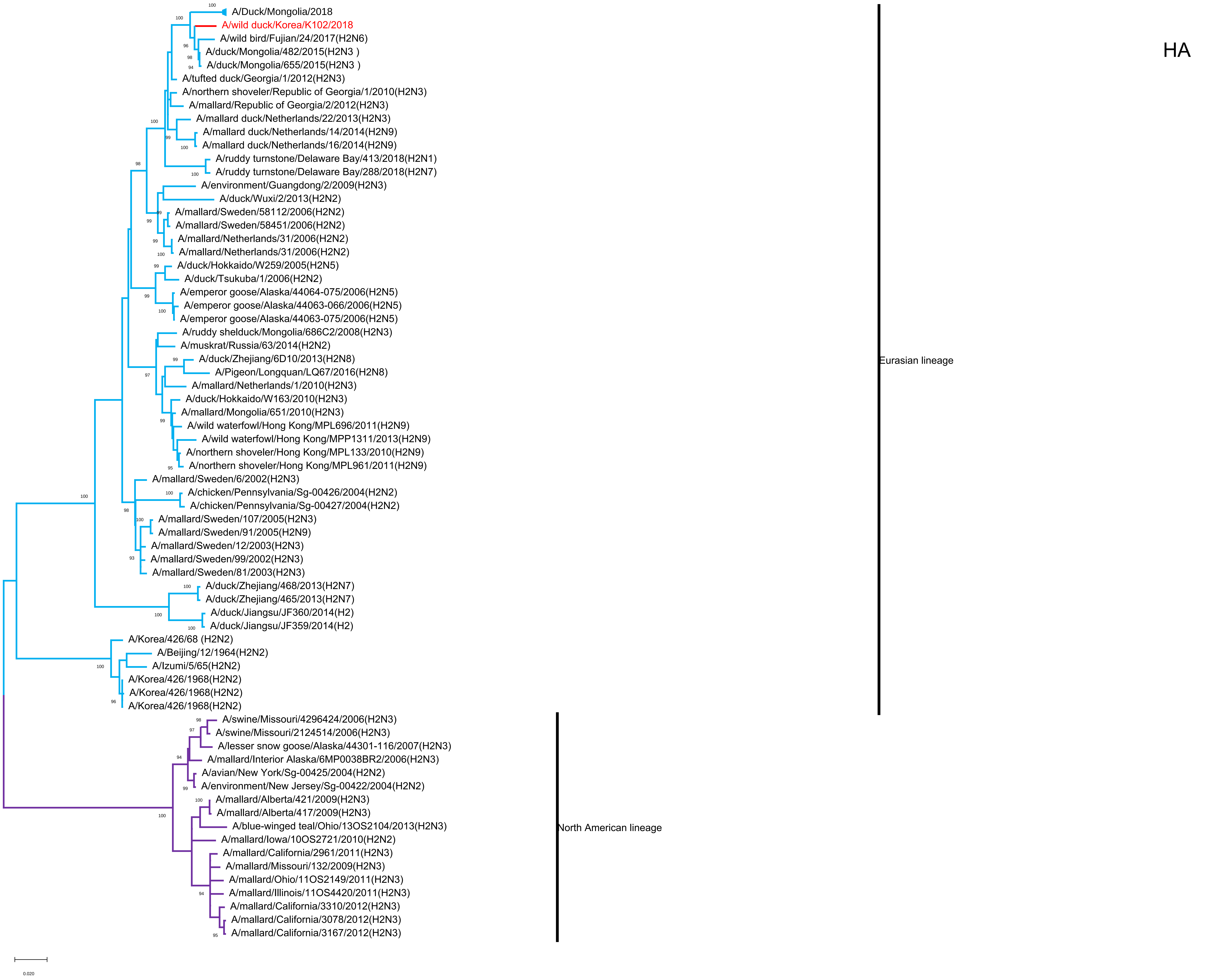
HA
A/Duck/Mongolia/2018
A/wild duck/Korea/K102/2018
A/wild bird/Fujian/24/2017(H2N6)
A/duck/Mongolia/482/2015(H2N3)
A/duck/Mongolia/655/2015(H2N3)
A/tufted duck/Georgia/1/2012(H2N3)
A/northern shoveler/Republic of Georgia/1/2010(H2N3)
A/mallard/Republic of Georgia/2/2012(H2N3)
A/mallard duck/Netherlands/22/2013(H2N3)
A/mallard duck/Netherlands/14/2014(H2N9)
A/mallard duck/Netherlands/16/2014(H2N9)
A/ruddy turnstone/Delaware Bay/413/2018(H2N1)
A/ruddy turnstone/Delaware Bay/288/2018(H2N7)
A/environment/Guangdong/2/2009(H2N3)
A/duck/Wuxi/2/2013(H2N2)
A/mallard/Sweden/58112/2006(H2N2)
A/mallard/Sweden/58451/2006(H2N2)
A/mallard/Netherlands/31/2006(H2N2)
A/mallard/Netherlands/31/2006(H2N2)
A/duck/Hokkaido/W259/2005(H2N5)
A/duck/Tsukuba/1/2006(H2N2)
A/emperor goose/Alaska/44064-075/2006(H2N5)
A/emperor goose/Alaska/44063-066/2006(H2N5)
A/emperor goose/Alaska/44063-075/2006(H2N5)
A/ruddy shelduck/Mongolia/686C2/2008(H2N3)
A/muskrat/Russia/63/2014(H2N2)
A/duck/Zhejiang/6D10/2013(H2N8)
A/Pigeon/Longquan/LQ67/2016(H2N8)
A/mallard/Netherlands/1/2010(H2N3)
A/duck/Hokkaido/W163/2010(H2N3)
A/mallard/Mongolia/651/2010(H2N3)
A/wild waterfowl/Hong Kong/MPL696/2011(H2N9)
A/wild waterfowl/Hong Kong/MPP1311/2013(H2N9)
A/northern shoveler/Hong Kong/MPL133/2010(H2N9)
A/northern shoveler/Hong Kong/MPL961/2011(H2N9)
A/mallard/Sweden/6/2002(H2N3)
A/chicken/Pennsylvania/Sg-00426/2004(H2N2)
A/chicken/Pennsylvania/Sg-00427/2004(H2N2)
A/mallard/Sweden/107/2005(H2N3)
A/mallard/Sweden/91/2005(H2N9)
A/mallard/Sweden/12/2003(H2N3)
A/mallard/Sweden/99/2002(H2N3)
A/mallard/Sweden/81/2003(H2N3)
A/duck/Zhejiang/468/2013(H2N7)
A/duck/Zhejiang/465/2013(H2N7)
A/duck/Jiangsu/JF360/2014(H2)
A/duck/Jiangsu/JF359/2014(H2)
A/Korea/426/68 (H2N2)
A/Beijing/12/1964(H2N2)
A/Izumi/5/65(H2N2)
A/Korea/426/1968(H2N2)
A/Korea/426/1968(H2N2)
A/Korea/426/1968(H2N2)
Eurasian lineage
A/swine/Missouri/4296424/2006(H2N3)
A/swine/Missouri/2124514/2006(H2N3)
A/lesser snow goose/Alaska/44301-116/2007(H2N3)
A/mallard/Interior Alaska/6MP0038BR2/2006(H2N3)
A/avian/New York/Sg-00425/2004(H2N2)
A/environment/New Jersey/Sg-00422/2004(H2N2)
A/mallard/Alberta/421/2009(H2N3)
A/mallard/Alberta/417/2009(H2N3)
A/blue-winged teal/Ohio/13OS2104/2013(H2N3)
A/mallard/Iowa/10OS2721/2010(H2N2)
A/mallard/California/2961/2011(H2N3)
A/mallard/Missouri/132/2009(H2N3)
A/mallard/Ohio/11OS2149/2011(H2N3)
A/mallard/Illinois/11OS4420/2011(H2N3)
A/mallard/California/3310/2012(H2N3)
A/mallard/California/3078/2012(H2N3)
A/mallard/California/3167/2012(H2N3)
North American lineage
0.020

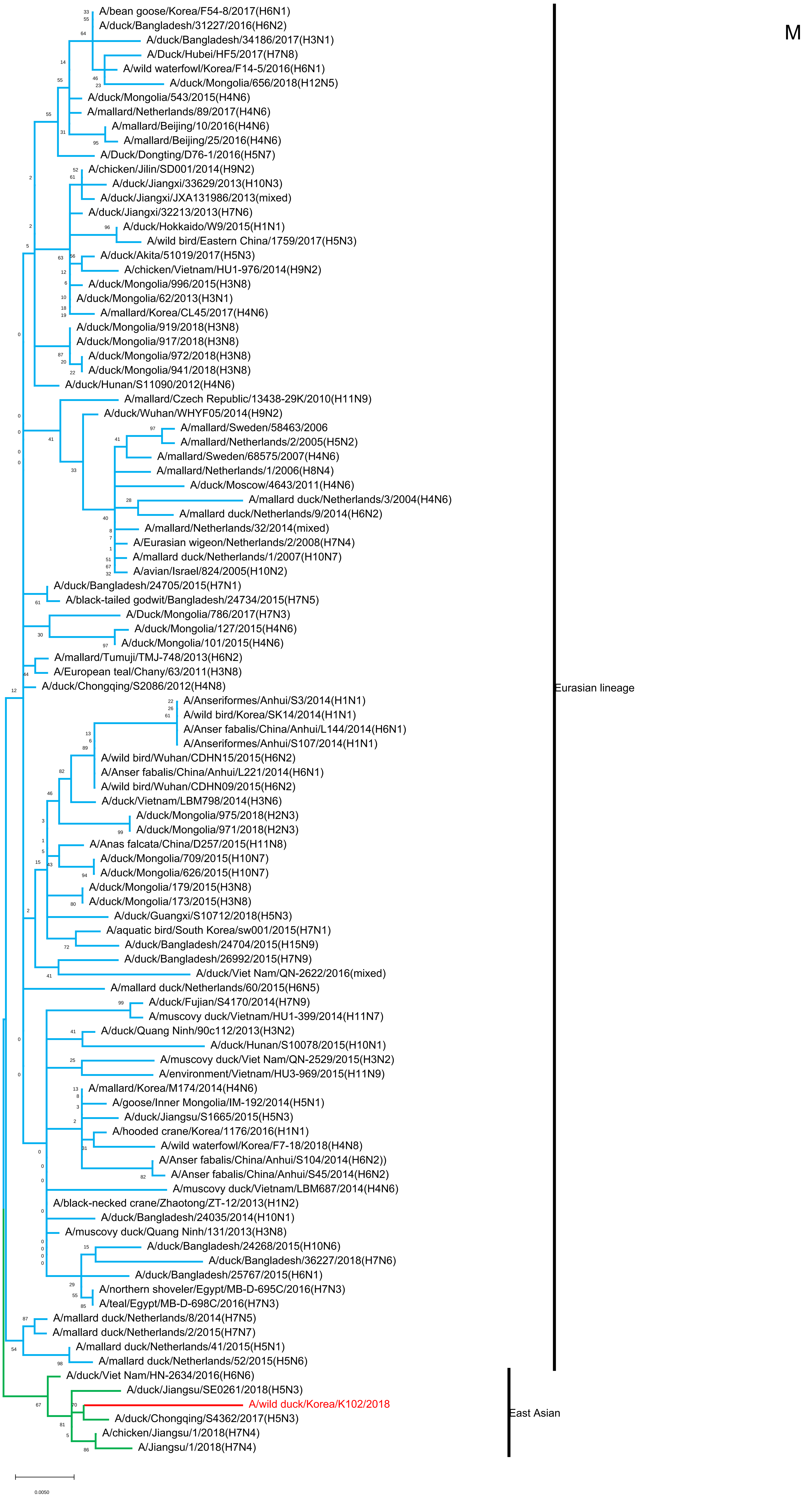

M
A/bean goose/Korea/F54-8/2017(H6N1)
A/duck/Bangladesh/31227/2016(H6N2)
A/duck/Bangladesh/34186/2017(H3N1)
A/Duck/Hubei/HF5/2017(H7N8)
A/wild waterfowl/Korea/F14-5/2016(H6N1)
A/duck/Mongolia/656/2018(H12N5)
A/duck/Mongolia/543/2015(H4N6)
A/mallard/Netherlands/89/2017(H4N6)
A/mallard/Beijing/10/2016(H4N6)
A/mallard/Beijing/25/2016(H4N6)
A/Duck/Dongting/D76-1/2016(H5N7)
A/chicken/Jilin/SD001/2014(H9N2)
A/duck/Jiangxi/33629/2013(H10N3)
A/duck/Jiangxi/JXA131986/2013(mixed)
A/duck/Jiangxi/32213/2013(H7N6)
A/duck/Hokkaido/W9/2015(H1N1)
A/wild bird/Eastern China/1759/2017(H5N3)
A/duck/Akita/51019/2017(H5N3)
A/chicken/Vietnam/HU1-976/2014(H9N2)
A/duck/Mongolia/996/2015(H3N8)
A/duck/Mongolia/62/2013(H3N1)
A/mallard/Korea/CL45/2017(H4N6)
A/duck/Mongolia/919/2018(H3N8)
A/duck/Mongolia/917/2018(H3N8)
A/duck/Mongolia/972/2018(H3N8)
A/duck/Mongolia/941/2018(H3N8)
A/duck/Hunan/S11090/2012(H4N6)
A/mallard/Czech Republic/13438-29K/2010(H11N9)
A/duck/Wuhan/WHYF05/2014(H9N2)
A/mallard/Sweden/58463/2006
A/mallard/Netherlands/2/2005(H5N2)
A/mallard/Sweden/68575/2007(H4N6)
A/mallard/Netherlands/1/2006(H8N4)
A/duck/Moscow/4643/2011(H4N6)
A/mallard duck/Netherlands/3/2004(H4N6)
A/mallard duck/Netherlands/9/2014(H6N2)
A/mallard/Netherlands/32/2014(mixed)
A/Eurasian wigeon/Netherlands/2/2008(H7N4)
A/mallard duck/Netherlands/1/2007(H10N7)
A/avian/Israel/824/2005(H10N2)
A/duck/Bangladesh/24705/2015(H7N1)
A/black-tailed godwit/Bangladesh/24734/2015(H7N5)
A/Duck/Mongolia/786/2017(H7N3)
A/duck/Mongolia/127/2015(H4N6)
A/duck/Mongolia/101/2015(H4N6)
A/mallard/Tumuji/TMJ-748/2013(H6N2)
A/European teal/Chany/63/2011(H3N8)
A/duck/Chongqing/S2086/2012(H4N8)
Eurasian lineage
A/Anseriformes/Anhui/S3/2014(H1N1)
A/wild bird/Korea/SK14/2014(H1N1)
A/Anser fabalis/China/Anhui/L144/2014(H6N1)
A/Anseriformes/Anhui/S107/2014(H1N1)
A/wild bird/Wuhan/CDHN15/2015(H6N2)
A/Anser fabalis/China/Anhui/L221/2014(H6N1)
A/wild bird/Wuhan/CDHN09/2015(H6N2)
A/duck/Vietnam/LBM798/2014(H3N6)
A/duck/Mongolia/975/2018(H2N3)
A/duck/Mongolia/971/2018(H2N3)
A/Anas falcata/China/D257/2015(H11N8)
A/duck/Mongolia/709/2015(H10N7)
A/duck/Mongolia/626/2015(H10N7)
A/duck/Mongolia/179/2015(H3N8)
A/duck/Mongolia/173/2015(H3N8)
A/duck/Guangxi/S10712/2018(H5N3)
A/aquatic bird/South Korea/sw001/2015(H7N1)
A/duck/Bangladesh/24704/2015(H15N9)
A/duck/Bangladesh/26992/2015(H7N9)
A/duck/Viet Nam/QN-2622/2016(mixed)
A/mallard duck/Netherlands/60/2015(H6N5)
A/duck/Fujian/S4170/2014(H7N9)
A/muscovy duck/Vietnam/HU1-399/2014(H11N7)
A/duck/Quang Ninh/90c112/2013(H3N2)
A/duck/Hunan/S10078/2015(H10N1)
A/muscovy duck/Viet Nam/QN-2529/2015(H3N2)
A/environment/Vietnam/HU3-969/2015(H11N9)
A/mallard/Korea/M174/2014(H4N6)
A/goose/Inner Mongolia/IM-192/2014(H5N1)
A/duck/Jiangsu/S1665/2015(H5N3)
A/hooded crane/Korea/1176/2016(H1N1)
A/wild waterfowl/Korea/F7-18/2018(H4N8)
A/Anser fabalis/China/Anhui/S104/2014(H6N2))
A/Anser fabalis/China/Anhui/S45/2014(H6N2)
A/muscovy duck/Vietnam/LBM687/2014(H4N6)
A/black-necked crane/Zhaotong/ZT-12/2013(H1N2)
A/duck/Bangladesh/24035/2014(H10N1)
A/muscovy duck/Quang Ninh/131/2013(H3N8)
A/duck/Bangladesh/24268/2015(H10N6)
A/duck/Bangladesh/36227/2018(H7N6)
A/duck/Bangladesh/25767/2015(H6N1)
A/northern shoveler/Egypt/MB-D-695C/2016(H7N3)
A/teal/Egypt/MB-D-698C/2016(H7N3)
A/mallard duck/Netherlands/8/2014(H7N5)
A/mallard duck/Netherlands/2/2015(H7N7)
A/mallard duck/Netherlands/41/2015(H5N1)
A/mallard duck/Netherlands/52/2015(H5N6)
A/duck/Viet Nam/HN-2634/2016(H6N6)
A/duck/Jiangsu/SE0261/2018(H5N3)
A/wild duck/Korea/K102/2018
A/duck/Chongqing/S4362/2017(H5N3)
A/chicken/Jiangsu/1/2018(H7N4)
A/Jiangsu/1/2018(H7N4)
East Asian
0.0050

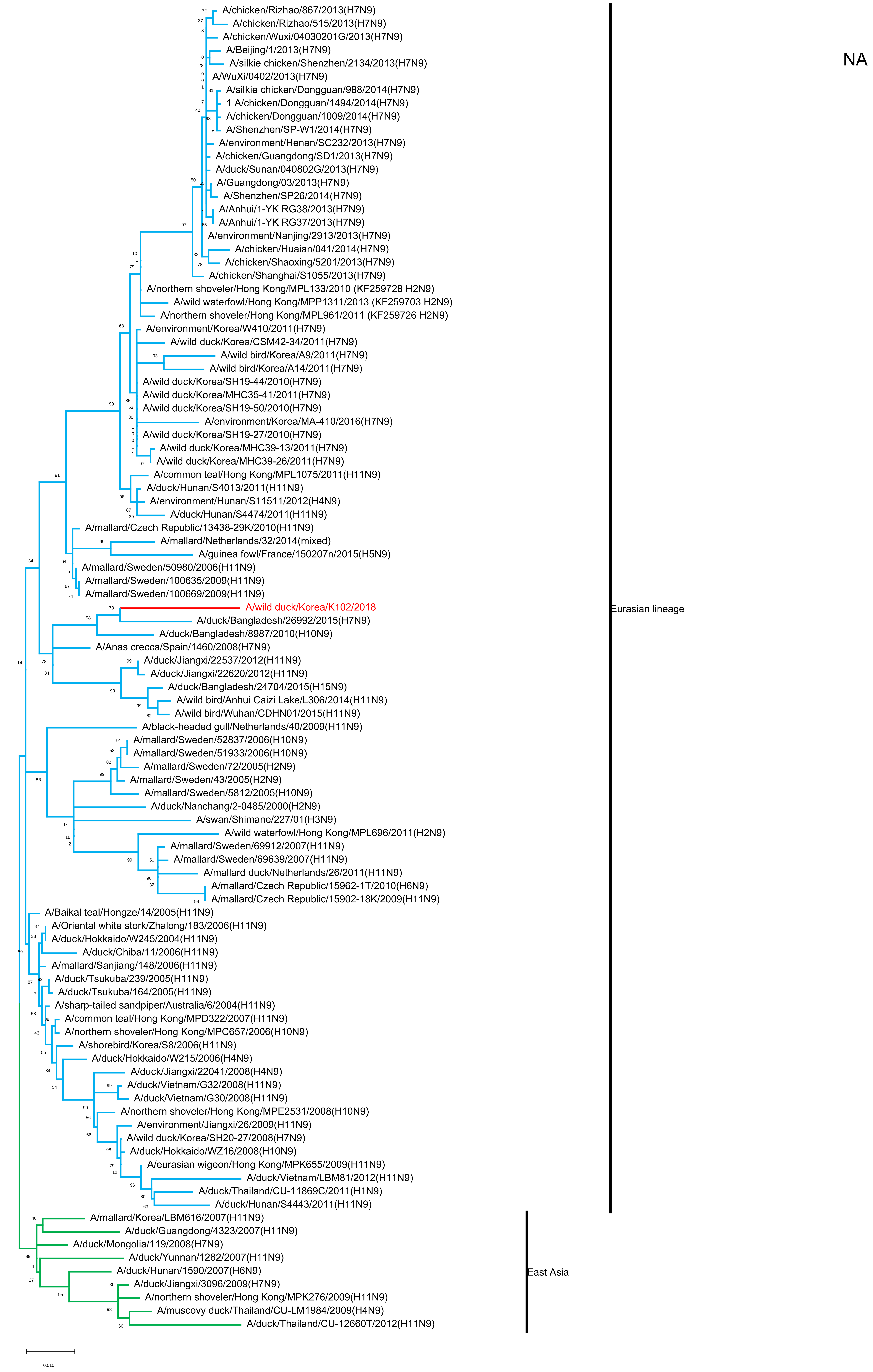
NA
A/chicken/Rizhao/867/2013(H7N9)
A/chicken/Rizhao/515/2013(H7N9)
A/chicken/Wuxi/04030201G/2013(H7N9)
A/Beijing/1/2013(H7N9)
A/silkie chicken/Shenzhen/2134/2013(H7N9)
A/WuXi/0402/2013(H7N9)
A/silkie chicken/Dongguan/988/2014(H7N9)
1 A/chicken/Dongguan/1494/2014(H7N9)
A/chicken/Dongguan/1009/2014(H7N9)
A/Shenzhen/SP-W1/2014(H7N9)
A/environment/Henan/SC232/2013(H7N9)
A/chicken/Guangdong/SD1/2013(H7N9)
A/duck/Sunan/040802G/2013(H7N9)
A/Guangdong/03/2013(H7N9)
A/Shenzhen/SP26/2014(H7N9)
A/Anhui/1-YK RG38/2013(H7N9)
A/Anhui/1-YK RG37/2013(H7N9)
A/environment/Nanjing/2913/2013(H7N9)
A/chicken/Huaian/041/2014(H7N9)
A/chicken/Shaoxing/5201/2013(H7N9)
A/chicken/Shanghai/S1055/2013(H7N9)
A/northern shoveler/Hong Kong/MPL133/2010 (KF259728 H2N9)
A/wild waterfowl/Hong Kong/MPP1311/2013 (KF259703 H2N9)
A/northern shoveler/Hong Kong/MPL961/2011 (KF259726 H2N9)
A/environment/Korea/W410/2011(H7N9)
A/wild duck/Korea/CSM42-34/2011(H7N9)
A/wild bird/Korea/A9/2011(H7N9)
A/wild bird/Korea/A14/2011(H7N9)
A/wild duck/Korea/SH19-44/2010(H7N9)
A/wild duck/Korea/MHC35-41/2011(H7N9)
A/wild duck/Korea/SH19-50/2010(H7N9)
A/environment/Korea/MA-410/2016(H7N9)
A/wild duck/Korea/SH19-27/2010(H7N9)
A/wild duck/Korea/MHC39-13/2011(H7N9)
A/wild duck/Korea/MHC39-26/2011(H7N9)
A/common teal/Hong Kong/MPL1075/2011(H11N9)
A/duck/Hunan/S4013/2011(H11N9)
A/environment/Hunan/S11511/2012(H4N9)
A/duck/Hunan/S4474/2011(H11N9)
A/mallard/Czech Republic/13438-29K/2010(H11N9)
A/mallard/Netherlands/32/2014(mixed)
A/guinea fowl/France/150207n/2015(H5N9)
A/mallard/Sweden/50980/2006(H11N9)
A/mallard/Sweden/100635/2009(H11N9)
A/mallard/Sweden/100669/2009(H11N9)
A/wild duck/Korea/K102/2018
A/duck/Bangladesh/26992/2015(H7N9)
A/duck/Bangladesh/8987/2010(H10N9)
A/Anas crecca/Spain/1460/2008(H7N9)
A/duck/Jiangxi/22537/2012(H11N9)
A/duck/Jiangxi/22620/2012(H11N9)
A/duck/Bangladesh/24704/2015(H15N9)
A/wild bird/Anhui Caizi Lake/L306/2014(H11N9)
A/wild bird/Wuhan/CDHN01/2015(H11N9)
A/black-headed gull/Netherlands/40/2009(H11N9)
A/mallard/Sweden/52837/2006(H10N9)
A/mallard/Sweden/51933/2006(H10N9)
A/mallard/Sweden/72/2005(H2N9)
A/mallard/Sweden/43/2005(H2N9)
A/mallard/Sweden/5812/2005(H10N9)
A/duck/Nanchang/2-0485/2000(H2N9)
A/swan/Shimane/227/01(H3N9)
A/wild waterfowl/Hong Kong/MPL696/2011(H2N9)
A/mallard/Sweden/69912/2007(H11N9)
A/mallard/Sweden/69639/2007(H11N9)
A/mallard duck/Netherlands/26/2011(H11N9)
A/mallard/Czech Republic/15962-1T/2010(H6N9)
A/mallard/Czech Republic/15902-18K/2009(H11N9)
A/Baikal teal/Hongze/14/2005(H11N9)
A/Oriental white stork/Zhalong/183/2006(H11N9)
A/duck/Hokkaido/W245/2004(H11N9)
A/duck/Chiba/11/2006(H11N9)
A/mallard/Sanjiang/148/2006(H11N9)
A/duck/Tsukuba/239/2005(H11N9)
A/duck/Tsukuba/164/2005(H11N9)
A/sharp-tailed sandpiper/Australia/6/2004(H11N9)
A/common teal/Hong Kong/MPD322/2007(H11N9)
A/northern shoveler/Hong Kong/MPC657/2006(H10N9)
A/shorebird/Korea/S8/2006(H11N9)
A/duck/Hokkaido/W215/2006(H4N9)
A/duck/Jiangxi/22041/2008(H4N9)
A/duck/Vietnam/G32/2008(H11N9)
A/duck/Vietnam/G30/2008(H11N9)
A/northern shoveler/Hong Kong/MPE2531/2008(H10N9)
A/environment/Jiangxi/26/2009(H11N9)
A/wild duck/Korea/SH20-27/2008(H7N9)
A/duck/Hokkaido/WZ16/2008(H10N9)
A/eurasian wigeon/Hong Kong/MPK655/2009(H11N9)
A/duck/Vietnam/LBM81/2012(H11N9)
A/duck/Thailand/CU-11869C/2011(H1N9)
A/duck/Hunan/S4443/2011(H11N9)
Eurasian lineage
A/mallard/Korea/LBM616/2007(H11N9)
A/duck/Guangdong/4323/2007(H11N9)
A/duck/Mongolia/119/2008(H7N9)
A/duck/Yunnan/1282/2007(H11N9)
A/duck/Hunan/1590/2007(H6N9)
A/duck/Jiangxi/3096/2009(H7N9)
A/northern shoveler/Hong Kong/MPK276/2009(H11N9)
A/muscovy duck/Thailand/CU-LM1984/2009(H4N9)
A/duck/Thailand/CU-12660T/2012(H11N9)
East Asia
0.010

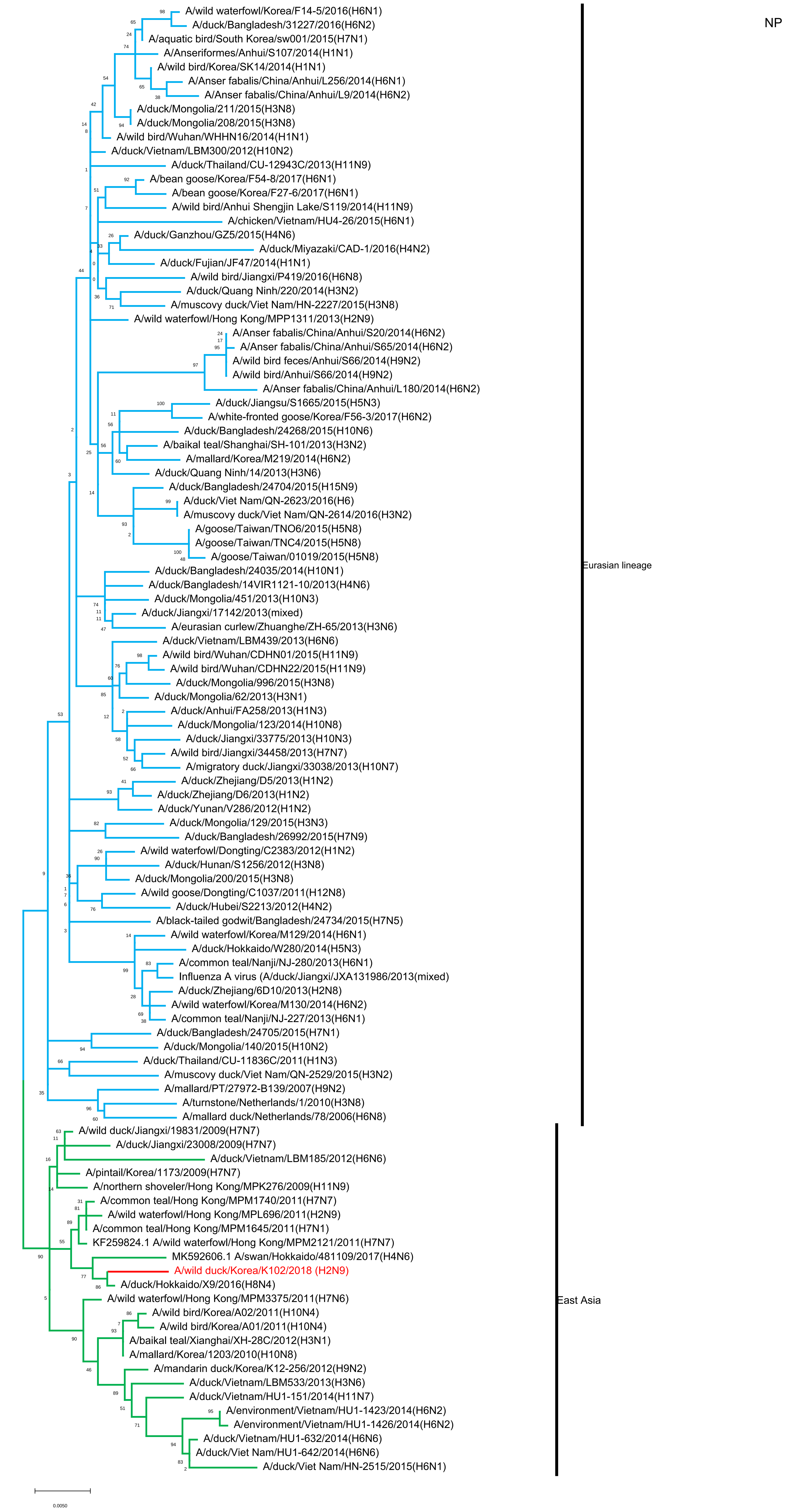
NP
A/wild waterfowl/Korea/F14-5/2016(H6N1)
A/duck/Bangladesh/31227/2016(H6N2)
A/aquatic bird/South Korea/sw001/2015(H7N1)
A/Anseriformes/Anhui/S107/2014(H1N1)
A/wild bird/Korea/SK14/2014(H1N1)
A/Anser fabalis/China/Anhui/L256/2014(H6N1)
A/Anser fabalis/China/Anhui/L9/2014(H6N2)
A/duck/Mongolia/211/2015(H3N8)
A/duck/Mongolia/208/2015(H3N8)
A/wild bird/Wuhan/WHHN16/2014(H1N1)
A/duck/Vietnam/LBM300/2012(H10N2)
A/duck/Thailand/CU-12943C/2013(H11N9)
A/bean goose/Korea/F54-8/2017(H6N1)
A/bean goose/Korea/F27-6/2017(H6N1)
A/wild bird/Anhui Shengjin Lake/S119/2014(H11N9)
A/chicken/Vietnam/HU4-26/2015(H6N1)
A/duck/Ganzhou/GZ5/2015(H4N6)
A/duck/Miyazaki/CAD-1/2016(H4N2)
A/duck/Fujian/JF47/2014(H1N1)
A/wild bird/Jiangxi/P419/2016(H6N8)
A/duck/Quang Ninh/220/2014(H3N2)
A/muscovy duck/Viet Nam/HN-2227/2015(H3N8)
A/wild waterfowl/Hong Kong/MPP1311/2013(H2N9)
A/Anser fabalis/China/Anhui/S20/2014(H6N2)
A/Anser fabalis/China/Anhui/S65/2014(H6N2)
A/wild bird feces/Anhui/S66/2014(H9N2)
A/wild bird/Anhui/S66/2014(H9N2)
A/Anser fabalis/China/Anhui/L180/2014(H6N2)
A/duck/Jiangsu/S1665/2015(H5N3)
A/white-fronted goose/Korea/F56-3/2017(H6N2)
A/duck/Bangladesh/24268/2015(H10N6)
A/baikal teal/Shanghai/SH-101/2013(H3N2)
A/mallard/Korea/M219/2014(H6N2)
A/duck/Quang Ninh/14/2013(H3N6)
A/duck/Bangladesh/24704/2015(H15N9)
A/duck/Viet Nam/QN-2623/2016(H6)
A/muscovy duck/Viet Nam/QN-2614/2016(H3N2)
A/goose/Taiwan/TNO6/2015(H5N8)
A/goose/Taiwan/TNC4/2015(H5N8)
A/goose/Taiwan/01019/2015(H5N8)
A/duck/Bangladesh/24035/2014(H10N1)
A/duck/Bangladesh/14VIR1121-10/2013(H4N6)
A/duck/Mongolia/451/2013(H10N3)
A/duck/Jiangxi/17142/2013(mixed)
A/eurasian curlew/Zhuanghe/ZH-65/2013(H3N6)
A/duck/Vietnam/LBM439/2013(H6N6)
A/wild bird/Wuhan/CDHN01/2015(H11N9)
A/wild bird/Wuhan/CDHN22/2015(H11N9)
A/duck/Mongolia/996/2015(H3N8)
A/duck/Mongolia/62/2013(H3N1)
A/duck/Anhui/FA258/2013(H1N3)
A/duck/Mongolia/123/2014(H10N8)
A/duck/Jiangxi/33775/2013(H10N3)
A/wild bird/Jiangxi/34458/2013(H7N7)
A/migratory duck/Jiangxi/33038/2013(H10N7)
A/duck/Zhejiang/D5/2013(H1N2)
A/duck/Zhejiang/D6/2013(H1N2)
A/duck/Yunan/V286/2012(H1N2)
A/duck/Mongolia/129/2015(H3N3)
A/duck/Bangladesh/26992/2015(H7N9)
A/wild waterfowl/Dongting/C2383/2012(H1N2)
A/duck/Hunan/S1256/2012(H3N8)
A/duck/Mongolia/200/2015(H3N8)
A/wild goose/Dongting/C1037/2011(H12N8)
A/duck/Hubei/S2213/2012(H4N2)
A/black-tailed godwit/Bangladesh/24734/2015(H7N5)
A/wild waterfowl/Korea/M129/2014(H6N1)
A/duck/Hokkaido/W280/2014(H5N3)
A/common teal/Nanji/NJ-280/2013(H6N1)
Influenza A virus (A/duck/Jiangxi/JXA131986/2013(mixed)
A/duck/Zhejiang/6D10/2013(H2N8)
A/wild waterfowl/Korea/M130/2014(H6N2)
A/common teal/Nanji/NJ-227/2013(H6N1)
A/duck/Bangladesh/24705/2015(H7N1)
A/duck/Mongolia/140/2015(H10N2)
A/duck/Thailand/CU-11836C/2011(H1N3)
A/muscovy duck/Viet Nam/QN-2529/2015(H3N2)
A/mallard/PT/27972-B139/2007(H9N2)
A/turnstone/Netherlands/1/2010(H3N8)
A/mallard duck/Netherlands/78/2006(H6N8)
Eurasian lineage
A/wild duck/Jiangxi/19831/2009(H7N7)
A/duck/Jiangxi/23008/2009(H7N7)
A/duck/Vietnam/LBM185/2012(H6N6)
A/pintail/Korea/1173/2009(H7N7)
A/northern shoveler/Hong Kong/MPK276/2009(H11N9)
A/common teal/Hong Kong/MPM1740/2011(H7N7)
A/wild waterfowl/Hong Kong/MPL696/2011(H2N9)
A/common teal/Hong Kong/MPM1645/2011(H7N1)
KF259824.1 A/wild waterfowl/Hong Kong/MPM2121/2011(H7N7)
MK592606.1 A/swan/Hokkaido/481109/2017(H4N6)
A/wild duck/Korea/K102/2018 (H2N9)
A/duck/Hokkaido/X9/2016(H8N4)
A/wild waterfowl/Hong Kong/MPM3375/2011(H7N6)
A/wild bird/Korea/A02/2011(H10N4)
A/wild bird/Korea/A01/2011(H10N4)
A/baikal teal/Xianghai/XH-28C/2012(H3N1)
A/mallard/Korea/1203/2010(H10N8)
A/mandarin duck/Korea/K12-256/2012(H9N2)
A/duck/Vietnam/LBM533/2013(H3N6)
A/duck/Vietnam/HU1-151/2014(H11N7)
A/environment/Vietnam/HU1-1423/2014(H6N2)
A/environment/Vietnam/HU1-1426/2014(H6N2)
A/duck/Vietnam/HU1-632/2014(H6N6)
A/duck/Viet Nam/HU1-642/2014(H6N6)
A/duck/Viet Nam/HN-2515/2015(H6N1)
East Asia
0.0050

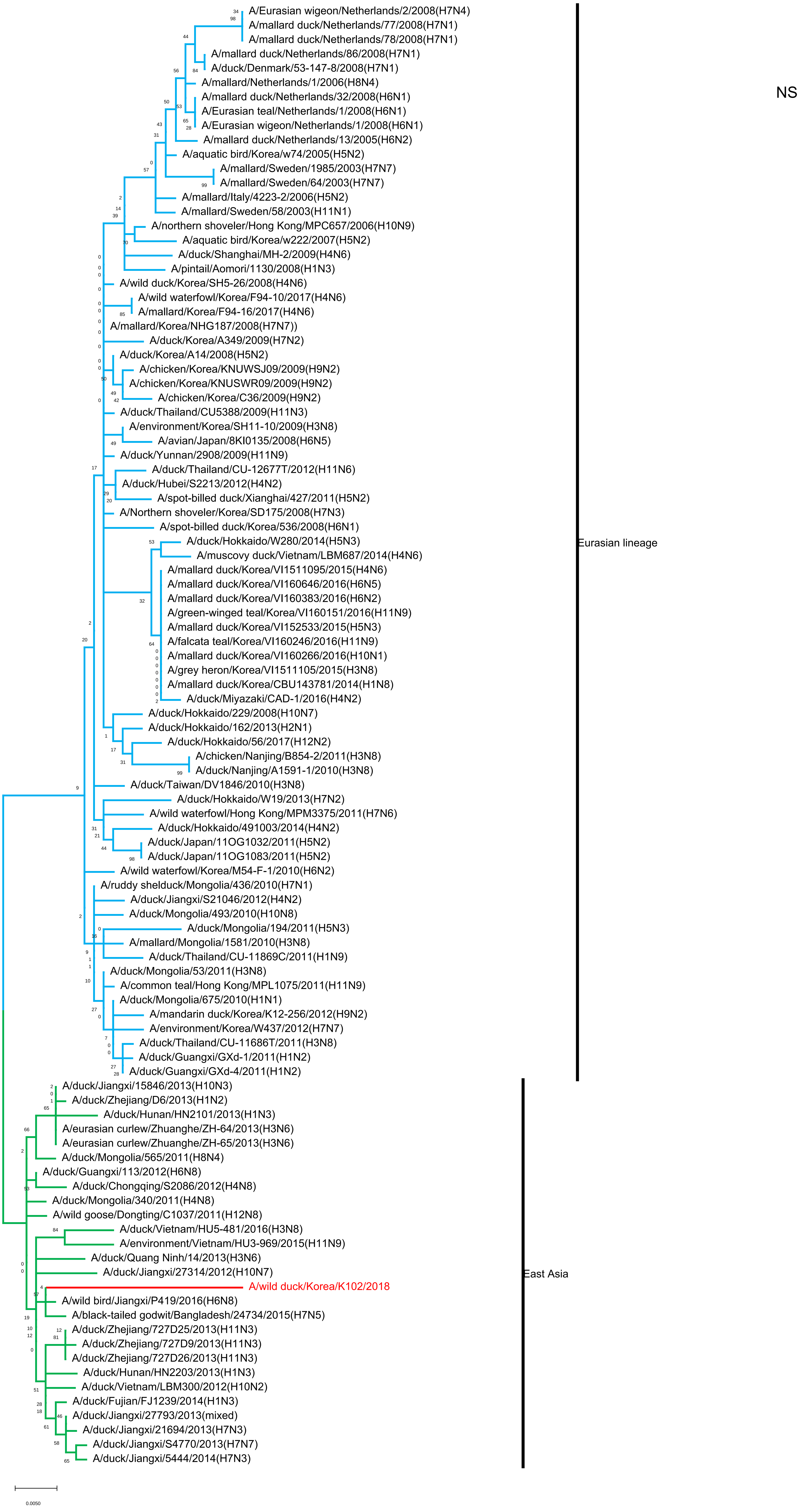
NS
A/Eurasian wigeon/Netherlands/2/2008(H7N4)
A/mallard duck/Netherlands/77/2008(H7N1)
A/mallard duck/Netherlands/78/2008(H7N1)
A/mallard duck/Netherlands/86/2008(H7N1)
A/duck/Denmark/53-147-8/2008(H7N1)
A/mallard/Netherlands/1/2006(H8N4)
A/mallard duck/Netherlands/32/2008(H6N1)
A/Eurasian teal/Netherlands/1/2008(H6N1)
A/Eurasian wigeon/Netherlands/1/2008(H6N1)
A/mallard duck/Netherlands/13/2005(H6N2)
A/aquatic bird/Korea/w74/2005(H5N2)
A/mallard/Sweden/1985/2003(H7N7)
A/mallard/Sweden/64/2003(H7N7)
A/mallard/Italy/4223-2/2006(H5N2)
A/mallard/Sweden/58/2003(H11N1)
A/northern shoveler/Hong Kong/MPC657/2006(H10N9)
A/aquatic bird/Korea/w222/2007(H5N2)
A/duck/Shanghai/MH-2/2009(H4N6)
A/pintail/Aomori/1130/2008(H1N3)
A/wild duck/Korea/SH5-26/2008(H4N6)
A/wild waterfowl/Korea/F94-10/2017(H4N6)
A/mallard/Korea/F94-16/2017(H4N6)
A/mallard/Korea/NHG187/2008(H7N7))
A/duck/Korea/A349/2009(H7N2)
A/duck/Korea/A14/2008(H5N2)
A/chicken/Korea/KNUWSJ09/2009(H9N2)
A/chicken/Korea/KNUSWR09/2009(H9N2)
A/chicken/Korea/C36/2009(H9N2)
A/duck/Thailand/CU5388/2009(H11N3)
A/environment/Korea/SH11-10/2009(H3N8)
A/avian/Japan/8KI0135/2008(H6N5)
A/duck/Yunnan/2908/2009(H11N9)
A/duck/Thailand/CU-12677T/2012(H11N6)
A/duck/Hubei/S2213/2012(H4N2)
A/spot-billed duck/Xianghai/427/2011(H5N2)
A/Northern shoveler/Korea/SD175/2008(H7N3)
A/spot-billed duck/Korea/536/2008(H6N1)
A/duck/Hokkaido/W280/2014(H5N3)
A/muscovy duck/Vietnam/LBM687/2014(H4N6)
A/mallard duck/Korea/VI1511095/2015(H4N6)
A/mallard duck/Korea/VI160646/2016(H6N5)
A/mallard duck/Korea/VI160383/2016(H6N2)
A/green-winged teal/Korea/VI160151/2016(H11N9)
A/mallard duck/Korea/VI152533/2015(H5N3)
A/falcata teal/Korea/VI160246/2016(H11N9)
A/mallard duck/Korea/VI160266/2016(H10N1)
A/grey heron/Korea/VI1511105/2015(H3N8)
A/mallard duck/Korea/CBU143781/2014(H1N8)
A/duck/Miyazaki/CAD-1/2016(H4N2)
A/duck/Hokkaido/229/2008(H10N7)
A/duck/Hokkaido/162/2013(H2N1)
A/duck/Hokkaido/56/2017(H12N2)
A/chicken/Nanjing/B854-2/2011(H3N8)
A/duck/Nanjing/A1591-1/2010(H3N8)
A/duck/Taiwan/DV1846/2010(H3N8)
A/duck/Hokkaido/W19/2013(H7N2)
A/wild waterfowl/Hong Kong/MPM3375/2011(H7N6)
A/duck/Hokkaido/491003/2014(H4N2)
A/duck/Japan/11OG1032/2011(H5N2)
A/duck/Japan/11OG1083/2011(H5N2)
A/wild waterfowl/Korea/M54-F-1/2010(H6N2)
A/ruddy shelduck/Mongolia/436/2010(H7N1)
A/duck/Jiangxi/S21046/2012(H4N2)
A/duck/Mongolia/493/2010(H10N8)
A/duck/Mongolia/194/2011(H5N3)
A/mallard/Mongolia/1581/2010(H3N8)
A/duck/Thailand/CU-11869C/2011(H1N9)
A/duck/Mongolia/53/2011(H3N8)
A/common teal/Hong Kong/MPL1075/2011(H11N9)
A/duck/Mongolia/675/2010(H1N1)
A/mandarin duck/Korea/K12-256/2012(H9N2)
A/environment/Korea/W437/2012(H7N7)
A/duck/Thailand/CU-11686T/2011(H3N8)
A/duck/Guangxi/GXd-1/2011(H1N2)
A/duck/Guangxi/GXd-4/2011(H1N2)
Eurasian lineage
A/duck/Jiangxi/15846/2013(H10N3)
A/duck/Zhejiang/D6/2013(H1N2)
A/duck/Hunan/HN2101/2013(H1N3)
A/eurasian curlew/Zhuanghe/ZH-64/2013(H3N6)
A/eurasian curlew/Zhuanghe/ZH-65/2013(H3N6)
A/duck/Mongolia/565/2011(H8N4)
A/duck/Guangxi/113/2012(H6N8)
A/duck/Chongqing/S2086/2012(H4N8)
A/duck/Mongolia/340/2011(H4N8)
A/wild goose/Dongting/C1037/2011(H12N8)
A/duck/Vietnam/HU5-481/2016(H3N8)
A/environment/Vietnam/HU3-969/2015(H11N9)
A/duck/Quang Ninh/14/2013(H3N6)
A/duck/Jiangxi/27314/2012(H10N7)
A/wild duck/Korea/K102/2018
A/wild bird/Jiangxi/P419/2016(H6N8)
A/black-tailed godwit/Bangladesh/24734/2015(H7N5)
A/duck/Zhejiang/727D25/2013(H11N3)
A/duck/Zhejiang/727D9/2013(H11N3)
A/duck/Zhejiang/727D26/2013(H11N3)
A/duck/Hunan/HN2203/2013(H1N3)
A/duck/Vietnam/LBM300/2012(H10N2)
A/duck/Fujian/FJ1239/2014(H1N3)
A/duck/Jiangxi/27793/2013(mixed)
A/duck/Jiangxi/21694/2013(H7N3)
A/duck/Jiangxi/S4770/2013(H7N7)
A/duck/Jiangxi/5444/2014(H7N3)
East Asia
0.0050

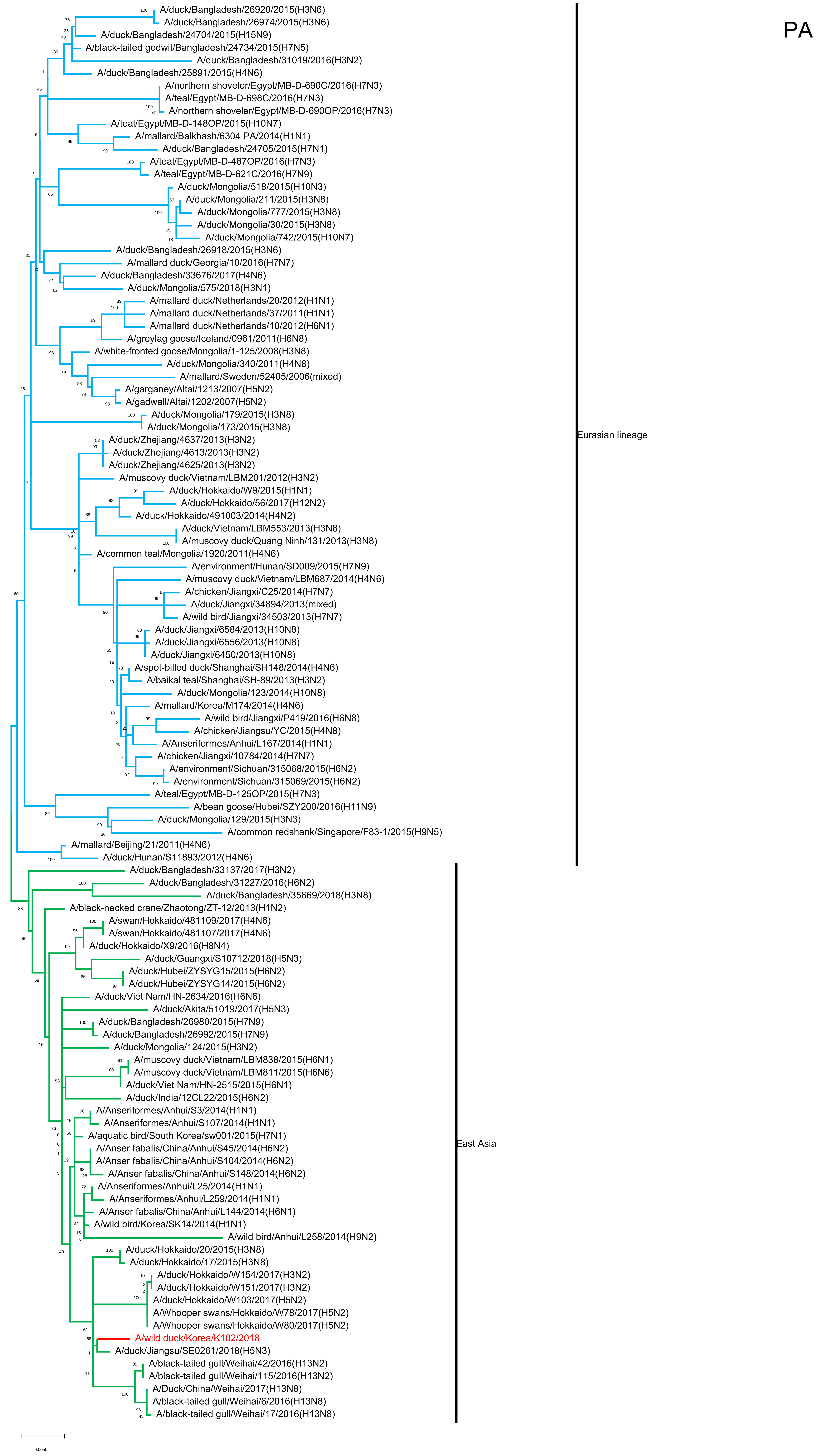

PA
A/duck/Bangladesh/26920/2015(H3N6)
A/duck/Bangladesh/26974/2015(H3N6)
A/duck/Bangladesh/24704/2015(H15N9)
A/black-tailed godwit/Bangladesh/24734/2015(H7N5)
A/duck/Bangladesh/31019/2016(H3N2)
A/duck/Bangladesh/25891/2015(H4N6)
A/northern shoveler/Egypt/MB-D-690C/2016(H7N3)
A/teal/Egypt/MB-D-698C/2016(H7N3)
A/northern shoveler/Egypt/MB-D-690OP/2016(H7N3)
A/teal/Egypt/MB-D-148OP/2015(H10N7)
A/mallard/Balkhash/6304 PA/2014(H1N1)
A/duck/Bangladesh/24705/2015(H7N1)
A/teal/Egypt/MB-D-487OP/2016(H7N3)
A/teal/Egypt/MB-D-621C/2016(H7N9)
A/duck/Mongolia/518/2015(H10N3)
A/duck/Mongolia/211/2015(H3N8)
A/duck/Mongolia/777/2015(H3N8)
A/duck/Mongolia/30/2015(H3N8)
A/duck/Mongolia/742/2015(H10N7)
A/duck/Bangladesh/26918/2015(H3N6)
A/mallard duck/Georgia/10/2016(H7N7)
A/duck/Bangladesh/33676/2017(H4N6)
A/duck/Mongolia/575/2018(H3N1)
A/mallard duck/Netherlands/20/2012(H1N1)
A/mallard duck/Netherlands/37/2011(H1N1)
A/mallard duck/Netherlands/10/2012(H6N1)
A/greylag goose/Iceland/0961/2011(H6N8)
A/white-fronted goose/Mongolia/1-125/2008(H3N8)
A/duck/Mongolia/340/2011(H4N8)
A/mallard/Sweden/52405/2006(mixed)
A/garganey/Altai/1213/2007(H5N2)
A/gadwall/Altai/1202/2007(H5N2)
A/duck/Mongolia/179/2015(H3N8)
A/duck/Mongolia/173/2015(H3N8)
A/duck/Zhejiang/4637/2013(H3N2)
A/duck/Zhejiang/4613/2013(H3N2)
A/duck/Zhejiang/4625/2013(H3N2)
A/muscovy duck/Vietnam/LBM201/2012(H3N2)
A/duck/Hokkaido/W9/2015(H1N1)
A/duck/Hokkaido/56/2017(H12N2)
A/duck/Hokkaido/491003/2014(H4N2)
A/duck/Vietnam/LBM553/2013(H3N8)
A/muscovy duck/Quang Ninh/131/2013(H3N8)
A/common teal/Mongolia/1920/2011(H4N6)
A/environment/Hunan/SD009/2015(H7N9)
A/muscovy duck/Vietnam/LBM687/2014(H4N6)
A/chicken/Jiangxi/C25/2014(H7N7)
A/duck/Jiangxi/34894/2013(mixed)
A/wild bird/Jiangxi/34503/2013(H7N7)
A/duck/Jiangxi/6584/2013(H10N8)
A/duck/Jiangxi/6556/2013(H10N8)
A/duck/Jiangxi/6450/2013(H10N8)
A/spot-billed duck/Shanghai/SH148/2014(H4N6)
A/baikal teal/Shanghai/SH-89/2013(H3N2)
A/duck/Mongolia/123/2014(H10N8)
A/mallard/Korea/M174/2014(H4N6)
A/wild bird/Jiangxi/P419/2016(H6N8)
A/chicken/Jiangsu/YC/2015(H4N8)
A/Anseriformes/Anhui/L167/2014(H1N1)
A/chicken/Jiangxi/10784/2014(H7N7)
A/environment/Sichuan/315068/2015(H6N2)
A/environment/Sichuan/315069/2015(H6N2)
A/teal/Egypt/MB-D-125OP/2015(H7N3)
A/bean goose/Hubei/SZY200/2016(H11N9)
A/duck/Mongolia/129/2015(H3N3)
A/common redshank/Singapore/F83-1/2015(H9N5)
A/mallard/Beijing/21/2011(H4N6)
A/duck/Hunan/S11893/2012(H4N6)
Eurasian lineage
A/duck/Bangladesh/33137/2017(H3N2)
A/duck/Bangladesh/31227/2016(H6N2)
A/duck/Bangladesh/35669/2018(H3N8)
A/black-necked crane/Zhaotong/ZT-12/2013(H1N2)
A/swan/Hokkaido/481109/2017(H4N6)
A/swan/Hokkaido/481107/2017(H4N6)
A/duck/Hokkaido/X9/2016(H8N4)
A/duck/Guangxi/S10712/2018(H5N3)
A/duck/Hubei/ZYSYG15/2015(H6N2)
A/duck/Hubei/ZYSYG14/2015(H6N2)
A/duck/Viet Nam/HN-2634/2016(H6N6)
A/duck/Akita/51019/2017(H5N3)
A/duck/Bangladesh/26980/2015(H7N9)
A/duck/Bangladesh/26992/2015(H7N9)
A/duck/Mongolia/124/2015(H3N2)
A/muscovy duck/Vietnam/LBM838/2015(H6N1)
A/muscovy duck/Vietnam/LBM811/2015(H6N6)
A/duck/Viet Nam/HN-2515/2015(H6N1)
A/duck/India/12CL22/2015(H6N2)
A/Anseriformes/Anhui/S3/2014(H1N1)
A/Anseriformes/Anhui/S107/2014(H1N1)
A/aquatic bird/South Korea/sw001/2015(H7N1)
A/Anser fabalis/China/Anhui/S45/2014(H6N2)
A/Anser fabalis/China/Anhui/S104/2014(H6N2)
A/Anser fabalis/China/Anhui/S148/2014(H6N2)
A/Anseriformes/Anhui/L25/2014(H1N1)
A/Anseriformes/Anhui/L259/2014(H1N1)
A/Anser fabalis/China/Anhui/L144/2014(H6N1)
A/wild bird/Korea/SK14/2014(H1N1)
A/wild bird/Anhui/L258/2014(H9N2)
East Asia
A/duck/Hokkaido/20/2015(H3N8)
A/duck/Hokkaido/17/2015(H3N8)
A/duck/Hokkaido/W154/2017(H3N2)
A/duck/Hokkaido/W151/2017(H3N2)
A/duck/Hokkaido/W103/2017(H5N2)
A/Whooper swans/Hokkaido/W78/2017(H5N2)
A/Whooper swans/Hokkaido/W80/2017(H5N2)
A/wild duck/Korea/K102/2018
A/duck/Jiangsu/SE0261/2018(H5N3)
A/black-tailed gull/Weihai/42/2016(H13N2)
A/black-tailed gull/Weihai/115/2016(H13N2)
A/Duck/China/Weihai/2017(H13N8)
A/black-tailed gull/Weihai/6/2016(H13N8)
A/black-tailed gull/Weihai/17/2016(H13N8)
0.0050

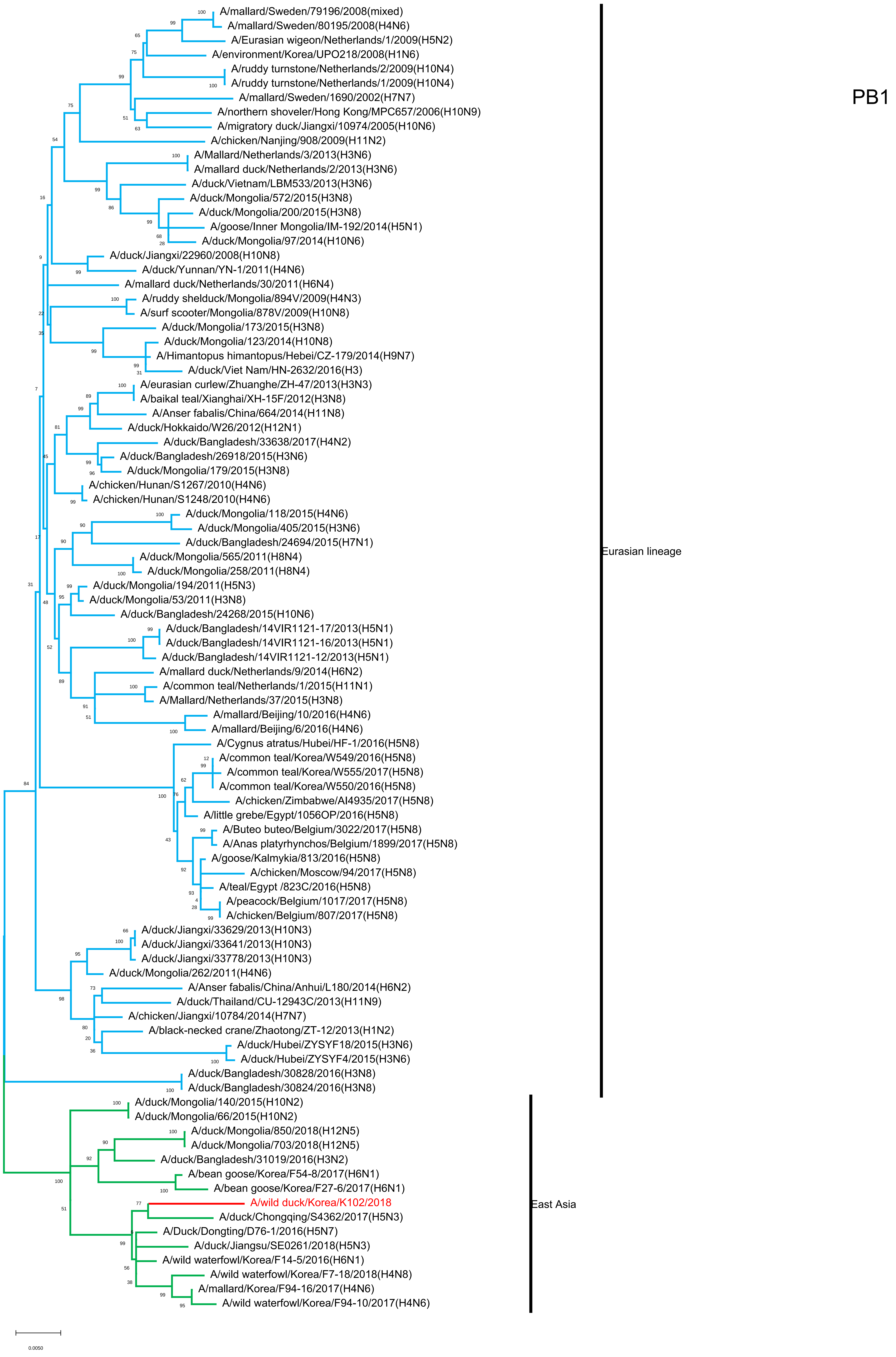
PB1
A/mallard/Sweden/79196/2008(mixed)
A/mallard/Sweden/80195/2008(H4N6)
A/Eurasian wigeon/Netherlands/1/2009(H5N2)
A/environment/Korea/UPO218/2008(H1N6)
A/ruddy turnstone/Netherlands/2/2009(H10N4)
A/ruddy turnstone/Netherlands/1/2009(H10N4)
A/mallard/Sweden/1690/2002(H7N7)
A/northern shoveler/Hong Kong/MPC657/2006(H10N9)
A/migratory duck/Jiangxi/10974/2005(H10N6)
A/chicken/Nanjing/908/2009(H11N2)
A/Mallard/Netherlands/3/2013(H3N6)
A/mallard duck/Netherlands/2/2013(H3N6)
A/duck/Vietnam/LBM533/2013(H3N6)
A/duck/Mongolia/572/2015(H3N8)
A/duck/Mongolia/200/2015(H3N8)
A/goose/Inner Mongolia/IM-192/2014(H5N1)
A/duck/Mongolia/97/2014(H10N6)
A/duck/Jiangxi/22960/2008(H10N8)
A/duck/Yunnan/YN-1/2011(H4N6)
A/mallard duck/Netherlands/30/2011(H6N4)
A/ruddy shelduck/Mongolia/894V/2009(H4N3)
A/surf scooter/Mongolia/878V/2009(H10N8)
A/duck/Mongolia/173/2015(H3N8)
A/duck/Mongolia/123/2014(H10N8)
A/Himantopus himantopus/Hebei/CZ-179/2014(H9N7)
A/duck/Viet Nam/HN-2632/2016(H3)
A/eurasian curlew/Zhuanghe/ZH-47/2013(H3N3)
A/baikal teal/Xianghai/XH-15F/2012(H3N8)
A/Anser fabalis/China/664/2014(H11N8)
A/duck/Hokkaido/W26/2012(H12N1)
A/duck/Bangladesh/33638/2017(H4N2)
A/duck/Bangladesh/26918/2015(H3N6)
A/duck/Mongolia/179/2015(H3N8)
A/chicken/Hunan/S1267/2010(H4N6)
A/chicken/Hunan/S1248/2010(H4N6)
A/duck/Mongolia/118/2015(H4N6)
A/duck/Mongolia/405/2015(H3N6)
A/duck/Bangladesh/24694/2015(H7N1)
A/duck/Mongolia/565/2011(H8N4)
A/duck/Mongolia/258/2011(H8N4)
A/duck/Mongolia/194/2011(H5N3)
A/duck/Mongolia/53/2011(H3N8)
A/duck/Bangladesh/24268/2015(H10N6)
A/duck/Bangladesh/14VIR1121-17/2013(H5N1)
A/duck/Bangladesh/14VIR1121-16/2013(H5N1)
A/duck/Bangladesh/14VIR1121-12/2013(H5N1)
A/mallard duck/Netherlands/9/2014(H6N2)
A/common teal/Netherlands/1/2015(H11N1)
A/Mallard/Netherlands/37/2015(H3N8)
A/mallard/Beijing/10/2016(H4N6)
A/mallard/Beijing/6/2016(H4N6)
A/Cygnus atratus/Hubei/HF-1/2016(H5N8)
A/common teal/Korea/W549/2016(H5N8)
A/common teal/Korea/W555/2017(H5N8)
A/common teal/Korea/W550/2016(H5N8)
A/chicken/Zimbabwe/AI4935/2017(H5N8)
A/little grebe/Egypt/1056OP/2016(H5N8)
A/Buteo buteo/Belgium/3022/2017(H5N8)
A/Anas platyrhynchos/Belgium/1899/2017(H5N8)
A/goose/Kalmykia/813/2016(H5N8)
A/chicken/Moscow/94/2017(H5N8)
A/teal/Egypt /823C/2016(H5N8)
A/peacock/Belgium/1017/2017(H5N8)
A/chicken/Belgium/807/2017(H5N8)
A/duck/Jiangxi/33629/2013(H10N3)
A/duck/Jiangxi/33641/2013(H10N3)
A/duck/Jiangxi/33778/2013(H10N3)
A/duck/Mongolia/262/2011(H4N6)
A/Anser fabalis/China/Anhui/L180/2014(H6N2)
A/duck/Thailand/CU-12943C/2013(H11N9)
A/chicken/Jiangxi/10784/2014(H7N7)
A/black-necked crane/Zhaotong/ZT-12/2013(H1N2)
A/duck/Hubei/ZYSYF18/2015(H3N6)
A/duck/Hubei/ZYSYF4/2015(H3N6)
A/duck/Bangladesh/30828/2016(H3N8)
A/duck/Bangladesh/30824/2016(H3N8)
Eurasian lineage
A/duck/Mongolia/140/2015(H10N2)
A/duck/Mongolia/66/2015(H10N2)
A/duck/Mongolia/850/2018(H12N5)
A/duck/Mongolia/703/2018(H12N5)
A/duck/Bangladesh/31019/2016(H3N2)
A/bean goose/Korea/F54-8/2017(H6N1)
A/bean goose/Korea/F27-6/2017(H6N1)
A/wild duck/Korea/K102/2018
A/duck/Chongqing/S4362/2017(H5N3)
A/Duck/Dongting/D76-1/2016(H5N7)
A/duck/Jiangsu/SE0261/2018(H5N3)
A/wild waterfowl/Korea/F14-5/2016(H6N1)
A/wild waterfowl/Korea/F7-18/2018(H4N8)
A/mallard/Korea/F94-16/2017(H4N6)
A/wild waterfowl/Korea/F94-10/2017(H4N6)
East Asia
0.0050

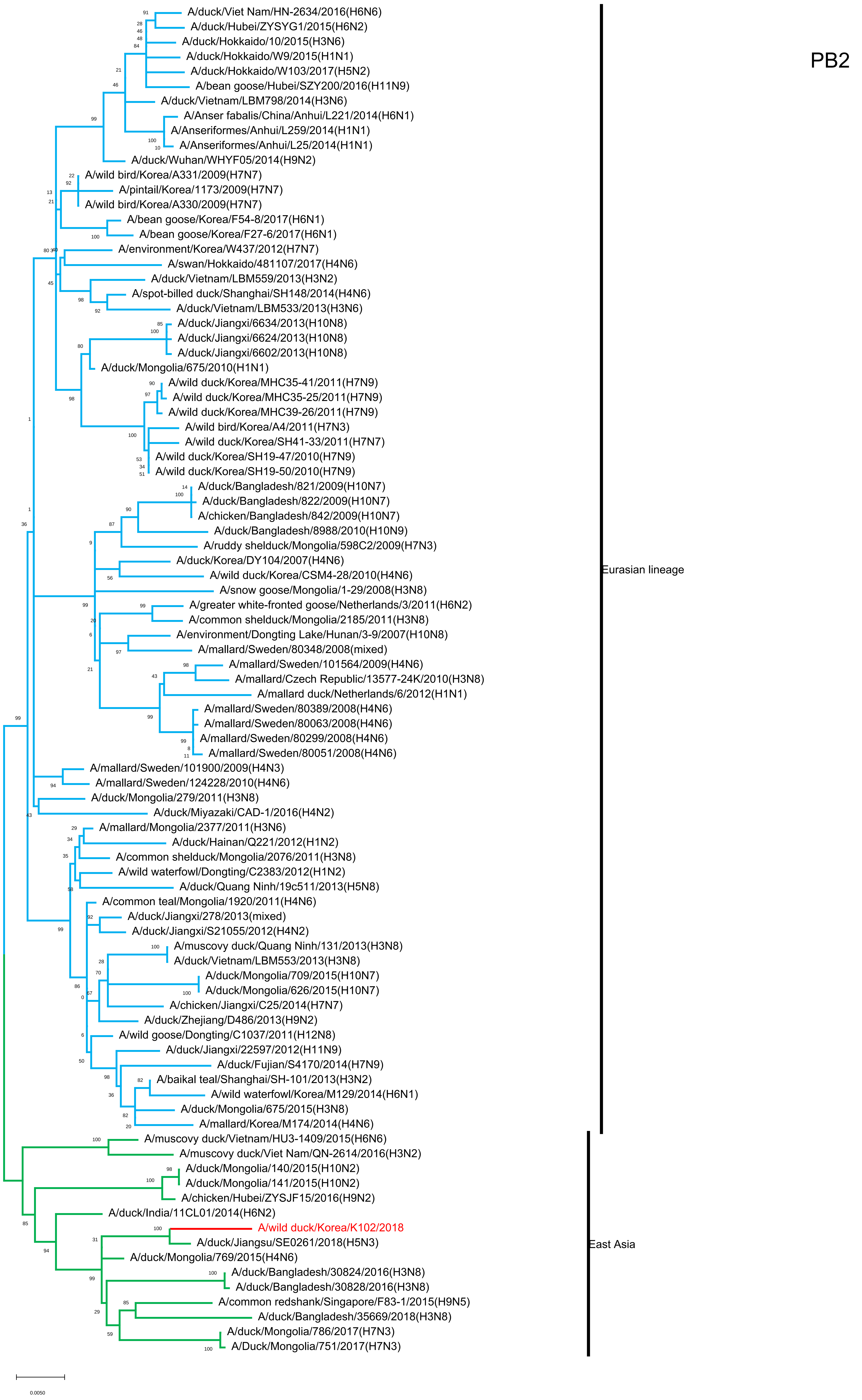
PB2
A/duck/Viet Nam/HN-2634/2016(H6N6)
A/duck/Hubei/ZYSYG1/2015(H6N2)
A/duck/Hokkaido/10/2015(H3N6)
A/duck/Hokkaido/W9/2015(H1N1)
A/duck/Hokkaido/W103/2017(H5N2)
A/bean goose/Hubei/SZY200/2016(H11N9)
A/duck/Vietnam/LBM798/2014(H3N6)
A/Anser fabalis/China/Anhui/L221/2014(H6N1)
A/Anseriformes/Anhui/L259/2014(H1N1)
A/Anseriformes/Anhui/L25/2014(H1N1)
A/duck/Wuhan/WHYF05/2014(H9N2)
A/wild bird/Korea/A331/2009(H7N7)
A/pintail/Korea/1173/2009(H7N7)
A/wild bird/Korea/A330/2009(H7N7)
A/bean goose/Korea/F54-8/2017(H6N1)
A/bean goose/Korea/F27-6/2017(H6N1)
A/environment/Korea/W437/2012(H7N7)
A/swan/Hokkaido/481107/2017(H4N6)
A/duck/Vietnam/LBM559/2013(H3N2)
A/spot-billed duck/Shanghai/SH148/2014(H4N6)
A/duck/Vietnam/LBM533/2013(H3N6)
A/duck/Jiangxi/6634/2013(H10N8)
A/duck/Jiangxi/6624/2013(H10N8)
A/duck/Jiangxi/6602/2013(H10N8)
A/duck/Mongolia/675/2010(H1N1)
A/wild duck/Korea/MHC35-41/2011(H7N9)
A/wild duck/Korea/MHC35-25/2011(H7N9)
A/wild duck/Korea/MHC39-26/2011(H7N9)
A/wild bird/Korea/A4/2011(H7N3)
A/wild duck/Korea/SH41-33/2011(H7N7)
A/wild duck/Korea/SH19-47/2010(H7N9)
A/wild duck/Korea/SH19-50/2010(H7N9)
A/duck/Bangladesh/821/2009(H10N7)
A/duck/Bangladesh/822/2009(H10N7)
A/chicken/Bangladesh/842/2009(H10N7)
A/duck/Bangladesh/8988/2010(H10N9)
A/ruddy shelduck/Mongolia/598C2/2009(H7N3)
A/duck/Korea/DY104/2007(H4N6)
A/wild duck/Korea/CSM4-28/2010(H4N6)
A/snow goose/Mongolia/1-29/2008(H3N8)
A/greater white-fronted goose/Netherlands/3/2011(H6N2)
A/common shelduck/Mongolia/2185/2011(H3N8)
A/environment/Dongting Lake/Hunan/3-9/2007(H10N8)
A/mallard/Sweden/80348/2008(mixed)
A/mallard/Sweden/101564/2009(H4N6)
A/mallard/Czech Republic/13577-24K/2010(H3N8)
A/mallard duck/Netherlands/6/2012(H1N1)
A/mallard/Sweden/80389/2008(H4N6)
A/mallard/Sweden/80063/2008(H4N6)
A/mallard/Sweden/80299/2008(H4N6)
A/mallard/Sweden/80051/2008(H4N6)
A/mallard/Sweden/101900/2009(H4N3)
A/mallard/Sweden/124228/2010(H4N6)
A/duck/Mongolia/279/2011(H3N8)
A/duck/Miyazaki/CAD-1/2016(H4N2)
A/mallard/Mongolia/2377/2011(H3N6)
A/duck/Hainan/Q221/2012(H1N2)
A/common shelduck/Mongolia/2076/2011(H3N8)
A/wild waterfowl/Dongting/C2383/2012(H1N2)
A/duck/Quang Ninh/19c511/2013(H5N8)
A/common teal/Mongolia/1920/2011(H4N6)
A/duck/Jiangxi/278/2013(mixed)
A/duck/Jiangxi/S21055/2012(H4N2)
A/muscovy duck/Quang Ninh/131/2013(H3N8)
A/duck/Vietnam/LBM553/2013(H3N8)
A/duck/Mongolia/709/2015(H10N7)
A/duck/Mongolia/626/2015(H10N7)
A/chicken/Jiangxi/C25/2014(H7N7)
A/duck/Zhejiang/D486/2013(H9N2)
A/wild goose/Dongting/C1037/2011(H12N8)
A/duck/Jiangxi/22597/2012(H11N9)
A/duck/Fujian/S4170/2014(H7N9)
A/baikal teal/Shanghai/SH-101/2013(H3N2)
A/wild waterfowl/Korea/M129/2014(H6N1)
A/duck/Mongolia/675/2015(H3N8)
A/mallard/Korea/M174/2014(H4N6)
Eurasian lineage
A/muscovy duck/Vietnam/HU3-1409/2015(H6N6)
A/muscovy duck/Viet Nam/QN-2614/2016(H3N2)
A/duck/Mongolia/140/2015(H10N2)
A/duck/Mongolia/141/2015(H10N2)
A/chicken/Hubei/ZYSJF15/2016(H9N2)
A/duck/India/11CL01/2014(H6N2)
A/wild duck/Korea/K102/2018
A/duck/Jiangsu/SE0261/2018(H5N3)
A/duck/Mongolia/769/2015(H4N6)
A/duck/Bangladesh/30824/2016(H3N8)
A/duck/Bangladesh/30828/2016(H3N8)
A/common redshank/Singapore/F83-1/2015(H9N5)
A/duck/Bangladesh/35669/2018(H3N8)
A/duck/Mongolia/786/2017(H7N3)
A/Duck/Mongolia/751/2017(H7N3)
East Asia
0.0050
